# Coexistence of specialist and generalist species within mixed plastic derivative-utilizing microbial communities

**DOI:** 10.1186/s40168-023-01645-4

**Published:** 2023-10-14

**Authors:** Laura Schaerer, Lindsay Putman, Isaac Bigcraft, Emma Byrne, Daniel Kulas, Ali Zolghadr, Sulihat Aloba, Rebecca Ong, David Shonnard, Stephen Techtmann

**Affiliations:** 1 Department of Biological Sciences, Michigan Technological University, 740 Dow ESE Building, 1400 Townsend Drive, Houghton, MI 49931 USA; 2 Department of Chemical Engineering, Michigan Technological University, Houghton, MI USA

**Keywords:** Polyethylene terephthalate, Upcycling, Biodegradation, Microbial community, Ecological interactions, Microbial community, Plastic upcycling, Specialist, Generalist, Biotechnology

## Abstract

**Background:**

Plastic-degrading microbial isolates offer great potential to degrade, transform, and upcycle plastic waste. Tandem chemical and biological processing of plastic wastes has been shown to substantially increase the rates of plastic degradation; however, the focus of this work has been almost entirely on microbial isolates (either bioengineered or naturally occurring). We propose that a microbial community has even greater potential for plastic upcycling. A microbial community has greater metabolic diversity to process mixed plastic waste streams and has built-in functional redundancy for optimal resilience.

**Results:**

Here, we used two plastic-derivative degrading communities as a model system to investigate the roles of specialist and generalist species within the microbial communities. These communities were grown on five plastic-derived substrates: pyrolysis treated high-density polyethylene, chemically deconstructed polyethylene terephthalate, disodium terephthalate, terephthalamide, and ethylene glycol. Short-read metagenomic and metatranscriptomic sequencing were performed to evaluate activity of microorganisms in each treatment. Long-read metagenomic sequencing was performed to obtain high-quality metagenome assembled genomes and evaluate division of labor.

**Conclusions:**

Data presented here show that the communities are primarily dominated by *Rhodococcus* generalists and lower abundance specialists for each of the plastic-derived substrates investigated here, supporting previous research that generalist species dominate batch culture. Additionally, division of labor may be present between *Hydrogenophaga* terephthalate degrading specialists and lower abundance protocatechuate degrading specialists﻿.

Video Abstract

**Supplementary Information:**

The online version contains supplementary material available at 10.1186/s40168-023-01645-4.

## Background

Many previous studies have demonstrated that chemical pre-processing of plastic waste followed by microbial biodegradation offers great promise for the development of future processes to upcycle post-consumer plastic waste into value-added products. Current methods for upcycling plastic requires the plastic to be sorted prior to processing, decreasing the efficiency of the process. An industrial upcycling process using a microbial community to metabolize mixed plastic could solve this problem. Here, we use microbial communities, which have been selectively enriched to degrade plastic derivatives, to investigate the ecological interactions of specialist and generalist species in microbial communities, including cooperation and competition. We hypothesize that (1) the microbial communities will be dominated by generalist species over specialist species and (2) the terephthalate degradation pathway will be shared by several microbial species through division of labor. This study demonstrates that ecological interactions such as these contribute to the microbial community’s ability to degrade a variety of plastic derivative substrates.

## Introduction

Over 70% of the 350 million tons of plastic produced each year pollutes the environment or ends up in landfills [1]. Alternative methods to current recycling practices are needed to limit the release of plastics to the environment and allow for conversion and upcycling of plastic waste. Recent research has demonstrated that microorganisms could be used to develop strategies for efficient management of plastic waste [2–4]. One strategy is to use microorganisms to upcycle waste plastic into more useful products. This often involves pairing chemical or thermal treatment with bioengineered microorganisms, which convert the carbon in the plastics to value-added products. Microbial isolates have been used to upcycle polyethylene terephthalate (PET) waste into the biodegradable plastic polyhydroxyalkanoate [5]. Similarly, bioengineered PETases have been used to upcycle PET into polyhydroxybutyrate (PHB) [6]. Guizik et al. showed that conversion of plastic into PHB can also be achieved with pyrolyzed polyethylene as a feedstock [7, 8]. Furthermore, Werner et al. showed that a nylon precursor (β-ketoadipic acid) could be produced from waste PET by feeding chemically deconstructed plastics to a bioengineered organism, *Pseudomonas putida* [9]. Another recent paper by Sullivan et al. showed that mixed plastics could be deconstructed using metal-catalyzed autoxidation and upcycled into valuable intermediate compounds using a bioengineered *Pseudomonas putida* strain [10]*.* These studies are only a few of many recent advances toward a circular plastic bioeconomy. However, the majority of these studies have focused their efforts on engineering bacterial isolates for consumption of deconstructed plastics and production of valuable commodity chemicals [11–13]. As the diversity of input streams increases, the need for engineering may increase the metabolic burden of these strains and decrease efficiency. Microbial communities provide flexibility in processing plastics wastes and synergistic interactions in communities can support efficient degradation of plastic wastes [14–16]. Here, we explore the mechanisms of community dynamics supporting flexible processing of thermal and chemically deconstructed plastics by two enriched microbial consortia. A better understanding of these mechanisms will be valuable to inform the construction of future industrial systems for upcycling plastic waste into food and other value-added products [3, 8, 10, 17, 18].

Plastic-degrading bacteria have been isolated from various natural environments, including marine [12], soil [19], and sewage [20]. In addition, the enzymatic pathways for degradation, assimilation, and mineralization of several plastics, including PET [21], are well known. However, environmental rates of plastic assimilation and mineralization are slow [22]. Even under laboratory conditions biodegradation of plastics is prohibitively slow for industrial processes. However, recent studies have suggested that coupling a chemical depolymerization step with microbial degradation in an industrial system may be a feasible way to upcycle excess plastic [3, 9]. As tools for upcycling plastic become more efficient, more plastic waste could be valorized, hopefully limiting the amount of plastic waste that is released to the environment.

A bacterial community may be more effective than an isolate for degrading mixtures of compounds due to the presence of diverse metabolisms, which may lead to more efficient use of resources by a community [23]. Microbial communities have been used for centuries to facilitate industrial processes including wastewater treatment [24] and production of fermented foods [25, 26]. Although humans have been exploiting microbial metabolisms for centuries, a lot remains unknown about microbial community functioning and the importance of ecological interactions in microbial communities. Within communities there exists a specialist-generalist paradigm [27]. Species face an evolutionary trade-off between performing a few functions very well (“specialists”) or performing many functions poorly (“generalists”) [28]. Rombouts et al. define a microbial specialist as an organism that takes up one substrate and a microbial generalist as an organism that takes up multiple substrates [29]. Metabolic specialists fill a smaller niche space and have few options for metabolism; in contrast, metabolic generalists will fill a larger niche space, having many options for metabolism [27, 29, 30]. Because specialist species have a narrow resource utilization range, their metabolic burden is lower, allowing for higher peak growth rates [27, 31]. Conversely, generalist species have a wider niche utilization range and a higher metabolic burden, often resulting in lower growth rates [27, 31]. Therefore, specialist species are expected to have an advantage over generalist species, especially in natural environments with abiotic gradients where there are many niches that can be exploited [27]. Gravel et al. previously showed that assemblages consisting of specialists utilized resources more efficiently compared to complementary generalist assemblages [23]. However, investigations into microbial specialization within synthetic industrial systems has shown that these systems are dominated by generalist species [29, 32]. Rombouts et al. found that generalists outcompeted specialists in a flow-through bioreactor system containing a synthetic community degrading xylose and glucose; they hypothesized that the generalist was able to utilize both substrates simultaneously, removing the specialist’s niche [29].

Individual species within communities can engage in either cooperative interactions or competitive interactions. Competitive interactions occur when individual species fill overlapping niches and compete for the same resources. Cooperative relationships between microbial species occur when the microorganisms engage in a mutually beneficial relationship; cooperation can consist of metabolic cross-feeding, division of labor, or other mutually beneficial activities [33]. Recent publications have suggested that cooperation in microbial communities is rare in microorganisms [34, 35]; however, many of these conclusions are based on experiments using synthetic consortia assembled with isolated microorganisms. The process of isolating microorganisms selects for microbes that are able to grow independently. Isolations are thought to bias results toward competitive relationships and against naturally occurring auxotrophies and interdependencies [35]. Mathematical models have also been used to elucidate the effects of cooperation and competition on overall microbial community stability. Positive interactions have been shown to have an overall destabilizing effect on communities [36], supporting the observations of many studies using synthetic consortia assembled from microbial isolates. In contrast, many articles have also described positive interactions between microorganisms [14, 15, 37, 38]. Thus, additional investigations into ecological interactions within a variety of microbial communities are warranted to better understand microbial community functioning.

Cooperative interactions within an industrial microbial community offer many benefits to the overall process including increased stability, resilience, and resistance to perturbations [39, 40]. By cross-feeding, sharing metabolites, and maintaining stable conditions, microorganisms living in communities have a lot of potential for cooperation. Often, one primary degrader expresses the enzymatic degradation pathway while being supported in some way by “helper” organisms. Helper organisms may provide necessary metabolites or metabolizing byproducts which are inhibitory to the primary degraders. Tucci et al. reported a cooperative (syntrophic) interaction between hydrocarbon degraders and electroactive bacteria, offering promise for groundwater remediation [41]. Additionally, Qi et al. demonstrated that co-culturing a sulfamethoxazole primary degrader (*Nocardioides*) and supporting organisms (*Acidovorax* and *Sphingobium*) led to complete catabolism of sulfamethoxazole [42]. In 2018, it was estimated that 98% of sequenced microorganisms were auxotrophs which rely on metabolites produced by other cells, suggesting that cooperation between microorganisms may be essential for microbial community functioning [43].

Division of labor is a type of cooperation in which microorganisms perform distinct steps within an enzymatic pathway, often for the mutual benefit of the organisms involved [44]. If a single species expresses a long enzymatic pathway alone, there is a high metabolic burden, which may be reduced if the enzymatic pathway can be shared between more than one species [45]. Division of labor is also known to promote efficient resource utilization [39, 40]. Tsoi et al. built mathematical models to compare the metabolic burden on microbial isolates compared to microbial consortia and predicted that division of labor would decrease metabolic burden and increase productivity of communities relative to monocultures [39]. However, when division of labor occurs, there is a trade-off in efficiency since metabolites must be transported between cells [45, 46]. Thommes et al. constructed in silico genome-scale models which showed complex interactions between microorganisms, for example where the TCA cycle was shared between several species [46]. Additionally, a recent study engineered a co-culture of two strains of *P. putida* for upcycling of deconstructed PET [47]. This study demonstrated that division of labor in co-culture resulted in enhanced performance of the co-culture compared to the monoculture.﻿ In spite of these prior investigations, understanding of microbial syntrophies and division of labor in microbial communities remains poorly understood and is an area of ongoing research [46].

The objective of this paper is to investigate ecological interactions within a microbial community that has been naturally enriched to degrade plastic-derived substrates. To explore microbial communities in high resolution without introducing the biases associated with isolation and culture techniques, we are using microbial communities, which have been artificially selected from natural environments to degrade plastic derivatives. By using a community of microorganisms instead of isolates, we expect low-abundance specialists within the microbial community to increase the metabolic diversity of our system, allowing the user to process multiple types of plastic. Current upcycling of post-consumer plastic requires the plastic to be sorted, increasing time and cost of the process [48]. An industrial upcycling process using a microbial community to metabolize mixed plastic could solve this problem [49]. Here, we use these model communities to investigate the roles of specialists and generalists, cooperation, and competition in microbial communities using metagenomics and metatranscriptomics. We hypothesize that (1) the microbial communities will be dominated by generalist species over specialist species and (2) the terephthalate degradation pathway will be shared by several microbial species through division of labor.

## Results

### Growth of microbial consortia on plastic-derived substrates

**Fig. 1:**
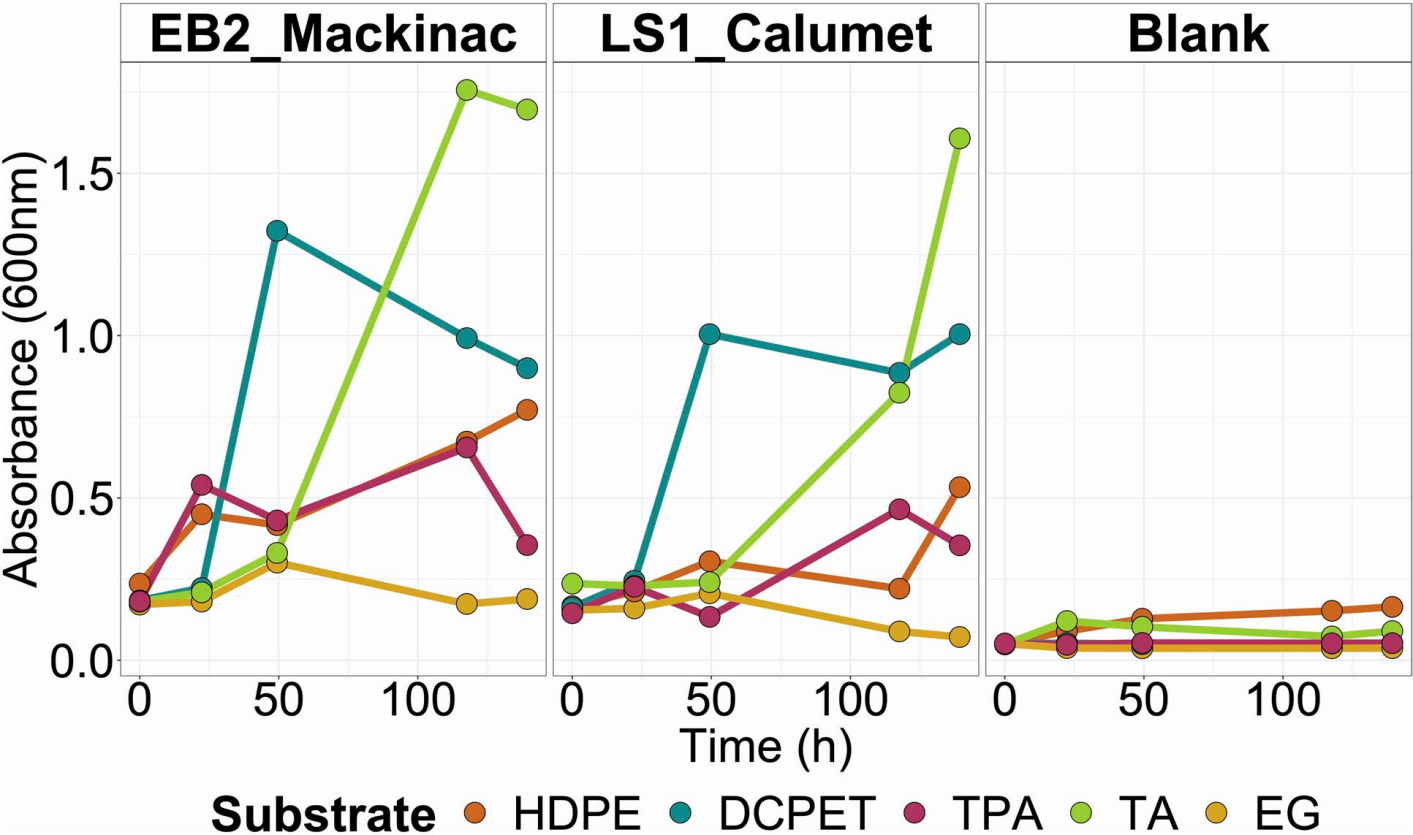
Growth of the two microbial communities (EB2_Mackinac and LS1_Calumet) on the five substrates used for this experiment: HDPE pyrolysis product, deconstructed PET (DCPET), terephthalate (TPA), terephthalamide (TA), and ethylene glycol (EG)

To demonstrate the flexibility of these consortia to grow on diverse deconstructed plastic substrates, we grew both consortia on pyrolysis-treated HDPE, deconstructed PET (DCPET), and the monomer compounds expected from the deconstruction of PET. The chemical composition of the chemically deconstructed PET was quantified with HPLC (Table S1). Both consortia, EB2_Mackinac and LS1_Calumet, were able to grow on different deconstructed plastic substrates. The extent of growth varied between the deconstructed plastic substrates. During the 136 h of growth, the change in OD_600_ (highest measured OD_600_ minus the lowest measured OD_600_) ranged between 0.129 and 1.576. The largest change in OD_600_ for the blank cultures was 0.114. Both microbial communities grew to the highest densities when grown on DCPET and terephthalamide; likewise, the lowest growth was seen in the ethylene glycol treatment for both microbial communities (Fig. 1, Table S2).

### Unique subsets of the community are active when grown on each substrate

**Fig. 2:**
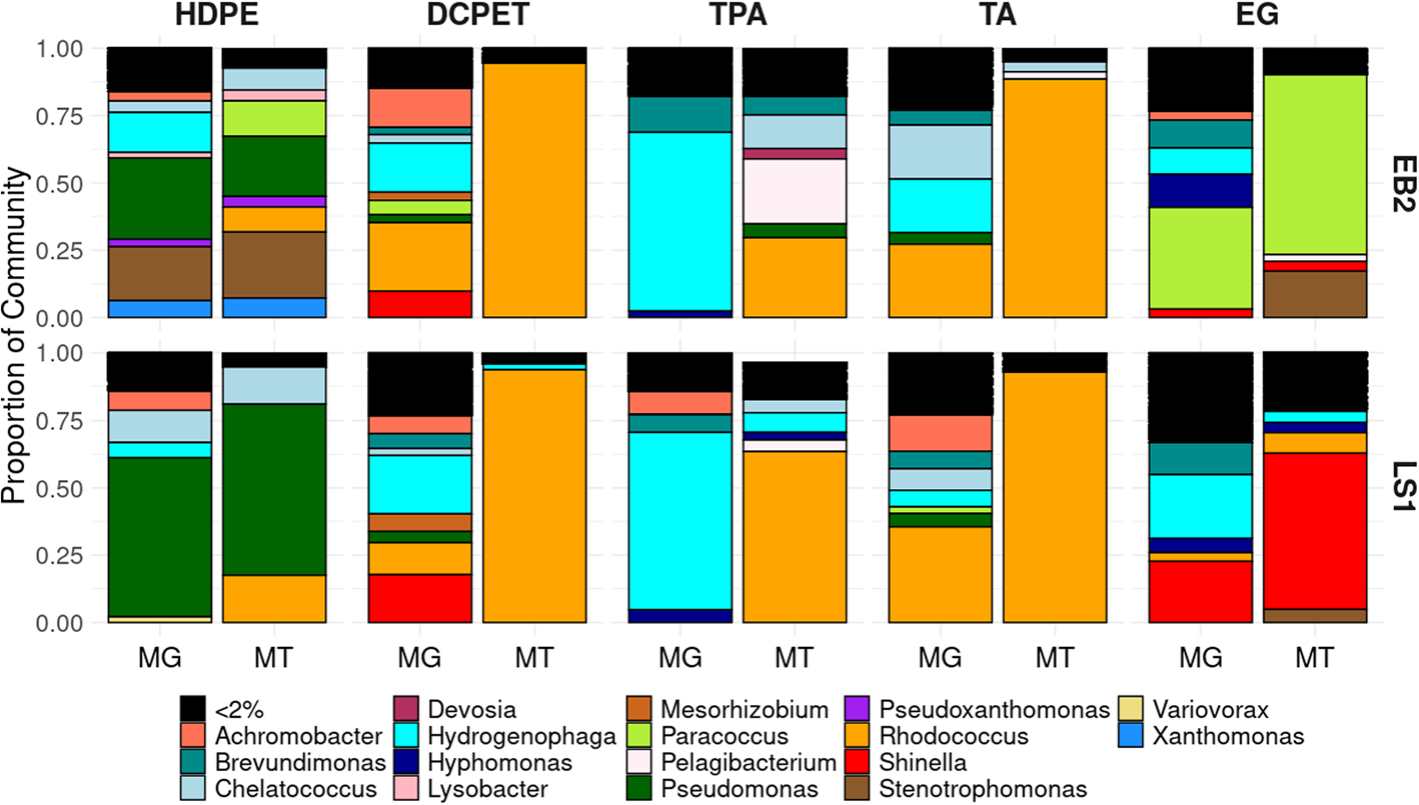
Microbial community composition of LS1_Calumet and EB2_Mackinac metagenomic (MG) and metatranscriptomic (MT) reads. Community composition shown for consortia grown on HDPE pyrolysis, deconstructed PET (DCPET), and PET-derivatives (TPA terephthalate, TA terephthalamide, EG ethylene glycol)

We assigned taxonomy to the metagenomics and metatranscriptomics reads to determine which microbial groups responded to different deconstructed plastic substrates. Short-read metagenomic and metatranscriptomic reads were analyzed and the taxonomy of each read was annotated with kraken2 (Fig. 2). The alpha diversity of metagenomic and metatranscriptomic reads was calculated for each sample using the metrics Observed and Shannon. Diversity of the EB2_Mackinac metagenomic samples ranged between 254 and 171 observed genera while the metatranscriptomic samples ranged between 137 and 55 observed genera (Fig. S1, Table S3). In the LS1_Calumet enrichment, the metagenomic samples ranged between 314 and 176 observed genera and the metatranscriptomic samples ranged between 156 and 44 observed genera (Fig. S1, Table S3). Overall, the metagenomic reads were more diverse and represented more genera compared to the metatranscriptomic reads. A Kruskal-Wallis test was used to test for statistically significant difference in alpha diversity between the sequence types (metagenomic and metatranscriptomic). A statistically significant difference was found for both Observed genera and Shannon alpha diversity metrics (*p* values < 0.001 and 0.003, respectively) (Table S4). This finding suggests that, while the microbial community is diverse, only a few members of the community are active on each substrate.

To visualize the differences between the community composition of the metagenomic communities and the active metatranscriptomic communities, a Bray Curtis distance matrix was constructed and a Principal Coordinate Analysis (PCoA) ordination was used to visualize differences in community composition between the metagenomic and metatranscriptomic samples (Fig. S3). The metagenomic samples clustered in the upper left corner of the plot, suggesting that all of the metagenomic samples have similar microbial community composition. A permutational analysis of variance (PERMANOVA) was performed to test for statistically significant differences in community composition between the metagenomic and metatranscriptomic samples, and this was found to be statistically significant (*p* value 0.003) (Table S5). On the lower left quadrant of the plot, the metatranscriptomic samples from cultures grown on ethylene glycol and HDPE pyrolysis loosely clustered together, suggesting that these substrates may be degraded by similar taxa. Likewise, the metatranscriptomic samples from cultures grown on terephthalate, terephthalamide, and chemically deconstructed PET cluster together in the upper right quadrant of the plot suggesting that similar taxa degrade these substrates. An additional PERMANOVA was performed to see if there was a statistically significant difference in the community composition of organisms transcriptomically active when grown on aromatic substrates (deconstructed PET, terephthalate, and terephthalamide) versus non-aromatic substrates (HDPE pyrolysis and ethylene glycol); this resulted in a statistically significant difference (*p* value 0.006) (Table S6). Interestingly, the terephthalate samples did not tightly cluster with the deconstructed PET and terephthalamide samples suggesting that there may be some unique differences in the community composition of the terephthalate degrading community compared to the deconstructed PET and terephthalamide treated samples.

### Communities are dominated by *Rhodococcus*

While the proportions of each organism vary between treatments, the metagenomic samples contain the same core taxa, supporting the observation on the PCoA plot suggesting that the metagenome samples all have similar community composition. All of the metagenomic samples have at least some contributions from *Rhodococcus* spp. for both EB2_Mackinac and LS1_Calumet enrichments, although the proportion is quite variable and ranges from < 1 to 65% (Fig. 2). *Hydrogenophaga* spp. are also present in all of the metagenomic samples for both enrichments, making up between 6 and 66% of the metagenomic reads in each treatment. Additionally, members of the genus *Pseudomonas* represented a substantial portion of the recovered metagenomic reads from both consortia when they were grown on HDPE. Other key taxa that make up large proportions of the metagenomic communities include members of the *Pseudomonas*, *Paracoccus*, *Chelatococcus*, and *Shinella* genera (Fig. 2, Table S7).

In the EB2_Mackinac community, the metagenomic reads were dominated by organisms from the genera *Hydrogenophaga*, *Pseudomonas*, *Stenotrophomonas*, *Achromobacter*, *Rhodococcus*, *Shinella*, *Hyphomonas*, and *Paracoccus*; the metatranscriptomic reads from EB2_Mackinac also showed that many of these taxa were most active when grown on plastic derivatives. When EB2_Mackinac was grown on HDPE pyrolysis, *Pseudomonas* (22% of all reads) and *Stenotrophomonas* (24% of all reads) were the most active genera. When EB2_mackinac was grown on DCPET and its individual components, *Rhodococcus* spp. were the most active organism on the full DCPET mixture (95% of all reads) as well as on terephthalate (30% of all reads) and terephthalamide (88% of all reads); when grown on ethylene glycol, *Paracoccus* was the most active organism (66% of all metatranscriptomic reads, compared to 38% of metagenomic reads). In the LS1_Calumet community, the metagenomic reads were comprised of representatives of the genera *Hydrogenophaga*, *Rhodococcus*, *Shinella*, *Brevundimonas*, and *Achromobacter*. The metatranscriptomic reads from LS1_Calumet showed that on HDPE pyrolysis *Pseudomonas* and *Rhodococcus* were most active. For both EB2_Mackinac and LS1_Calumet, *Rhodococcus* was remarkably more active than any other organism on the chemically deconstructed PET mixture (94.4% and 93.6%, respectively), terephthalate (30% and 64%, respectively), and terephthalamide (89% and 93%, respectively). On ethylene glycol, *Shinella* reads were more abundant in the metatranscriptomic reads (58%) compared to the metagenomic reads (28%).

Interestingly, the microbial communities from the terephthalate treatments for both enrichments had different composition than the chemically deconstructed PET and terephthalamide treatments. This was true for both the metagenomic and metatranscriptomic samples. The metagenomic communities from the terephthalate treatment for both enrichments were dominated by *Hydrogenophaga* (66% and 65%, respectively) as well as *Brevundimonas* and *Hyphomonas* (Fig. 2). The metatranscriptomic samples from the terephthalate treatments for both EB2_Mackinac and LS1_Calumet contain higher proportions of *Rhodococcus* (30% and 64%, respectively) relative to the metagenomic samples (both < 1%). Additionally, the proportion of *Hydrogenophaga* spp. was much lower in the metatranscriptomic samples relative to the metagenomic samples for both EB2_Mackinac (66 to 1.8%) and LS1_Calumet (65 to 7.1%). The terephthalate treatments were also the only treatments to have higher Shannon diversity of the metatranscriptomic reads compared to the metagenomic reads (Fig. S2, Table S3), indicating that the metatranscriptomic samples were less dominated by a single organism compared to the metagenomic samples. Using Kraken2 helps to identify active organisms based on the annotation of metatranscriptomic reads as belonging within a particular taxon. However, this annotation could be limited by the database that is being used as well as the accuracy of assigning taxonomy off of a short read.

### Specialist and generalist organisms

**Fig. 3:**
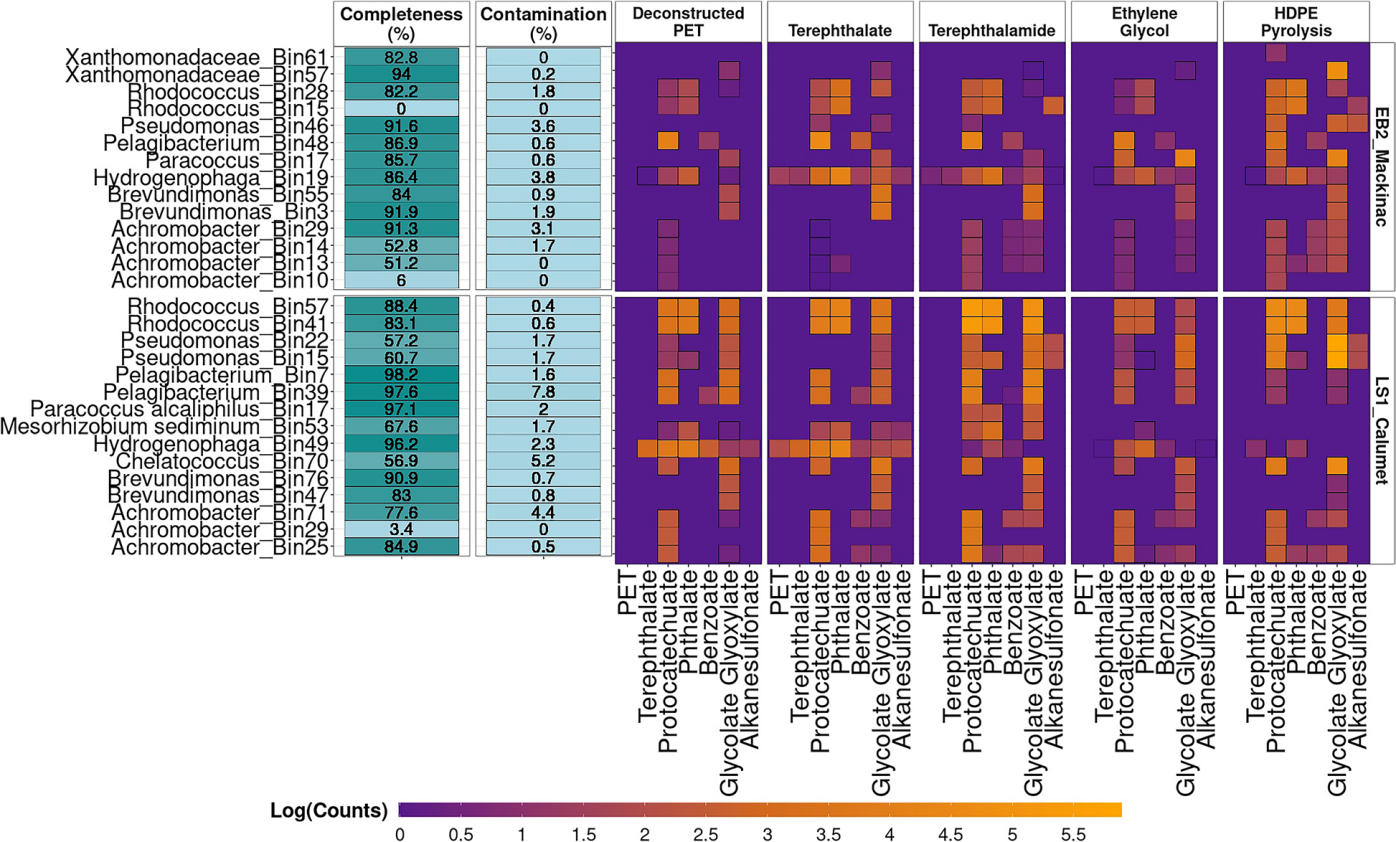
**A** Completeness and contamination percentages for MAGs obtained from short-read metagenomic sequencing. Low abundance MAGs are not shown here, see Fig. S5. **B** Gene expression of known aromatic, ethylene glycol, and hydrocarbon genes from each pathway. Abbreviations: DCPET (chemically deconstructed polyethylene terephthalate), TPA (terephthalate), TA (terephthalamide), EG (ethylene glycol), HDPE (high-density polyethylene pyrolysis)

To determine the activity of the organisms in each of the different treatments, we performed metatranscriptomic sequencing and mapped the metatranscriptomic reads to Metagenome Assembled Genomes (MAGs) generated from the short-read metagenomic sequencing (Illumina) described further in Table S10. This approach allows us to more confidently determine activity (expression of genes) of community members while having confidence in their taxonomy. Here, we specifically focus on the expression of pathways known to be involved in the degradation of aromatics (polyethylene terephthalate, phthalate, and benzoate), ethylene glycol, and hydrocarbons (alkanesulfonate monooxygenase) to infer cellular activity. The protocatechuate and glycolate/glyoxylate pathways were also investigated as central pathways, which are known to further degrade metabolites from protocatechuate and ethylene glycol, respectively, to fuel central metabolism. Results showed that the protocatechuate and glycolate/glyoxylate pathways were expressed by most MAGs across most treatments (Fig. 3B, Fig. S5B), many of the MAGs not expressing these genes were incomplete (Fig. 3A, Fig. S5A).

Genes known to be involved in polyethylene terephthalate and terephthalate degradation were only expressed by the *Hydrogenophaga* bins for both enrichments (HTSeq counts > 15) (Fig. 3, Fig. S5). These genes included the mono(2-hydroxyethyl) terephthalate hydrolase in both EB2_Mackinac and LS1_Calumet *Hydrogenophaga* MAGs. Additionally, the Hydrogenophaga strain from LS1_Calumet also expressed the genes for the terephthalate 1,2-dioxygenase alpha and beta subunits, as well as the reductase component. In the EB2_Mackinac enrichment, polyethylene terephthalate and terephthalate degrading genes were most highly expressed in the terephthalate and terephthalamide treatments. In the LS1_Calumet enrichment, the polyethylene terephthalate and terephthalate degrading genes were most highly expressed in the terephthalate and deconstructed PET treatments. Across the other pathways that were investigated here (protocatechuate, phthalate, and benzoate), the *Hydrogenophaga* MAGs from both enrichments showed the highest activity in the deconstructed PET, terephthalate, or terephthalamide treatments, with comparably much less activity in the ethylene glycol and HDPE treatments.

In the LS1_Calumet enrichment, the *Rhodococcus* MAGs were active across all treatments, particularly in the protocatechuate, phthalate, and glycolate/glyoxylate pathways (Fig. 3, Fig. S5). Interestingly, *Rhodococcus* appeared to be most active in the terephthalamide treatments relative to the other substrates. Similarly, in the EB2_Mackinac enrichment, *Rhodococcus* MAGs also expressed genes for the protocatechuate and phthalate pathways across most treatments, although at overall reduced levels compared to the LS1_Calumet enrichment.

Both *Pseudomonas* strains in the LS1_Calumet enrichment expressed alkanesulfonate monooxygenase genes in the HDPE treatment; interestingly, these *Pseudomonas* strains also expressed these genes in the terephthalamide treatment. In the LS1_Calumet enrichment, alkanesulfonate monooxygenase genes were also expressed by *Hydrogenophaga* in the deconstructed PET and terephthalate treatment and *Mesorhizobium sediminum* in the terephthalate treatment. Only one *Pseudomonas* strain was identified in the EB2_Mackinac enrichment. This strain also expressed the alkanesulfonate monooxygenase genes in the HDPE treatment. Additionally, one of the *Rhodococcus* strains from EB2_Mackinac also expressed alkanesulfonate monooxygenase genes in the HDPE and terephthalamide treatments.

### Division of labor between *Hydrogenophaga* terephthalate specialists and various protocatechuate specialists

To test the hypothesis that division of labor exists in these microbial communities, we used long-read sequencing. Long-read sequencing allows for closure of microbial genomes and thus would provide a more complete picture of the genetic content of the organisms in our community. The recovered MAGs are described in Table S9. We are defining division of labor in these communities as organisms that have fragmented pathways for degradation of deconstructed plastic substrates. Therefore, incomplete genomes could lead to incorrectly identifying an organism as having an incomplete biochemical pathway. To determine which taxa have complete pathways for the degradation of the various plastic derivative substrates, long-read sequencing was performed. Overall, MAGs recovered from long-read metagenomic sequencing had lower contamination, were more complete, and contained fewer contigs compared to the MAGs recovered from short-read metagenomic sequencing (Fig. S4, Table S8). From the EB2_Mackinac enrichment, 12 MAGs were obtained from PacBio sequencing and 31 MAGs were obtained from Illumina sequencing (less than 10% contaminated and greater than 70% complete, as determined by CheckM). The number of contigs in the PacBio MAGs ranged from 1 to 6, while the number of contigs in the Illumina MAGs ranged from 9 to 654. The median completeness for the PacBio MAGs was 98.6%, while the median completeness of the Illumina MAGs was only 86.4%. From the LS1_Calumet enrichment, 41 MAGs were obtained from PacBio sequencing, while 45 MAGs were obtained from Illumina sequencing. The number of contigs in the PacBio MAGs ranged from 1 to 34 contigs, while the number of contigs in the Illumina bins ranged from 4 to 553 contigs. The median completeness of the PacBio MAGs was 99.4%, while the median completeness of the Illumina MAGs was only 87.4%.

**Fig. 4:**
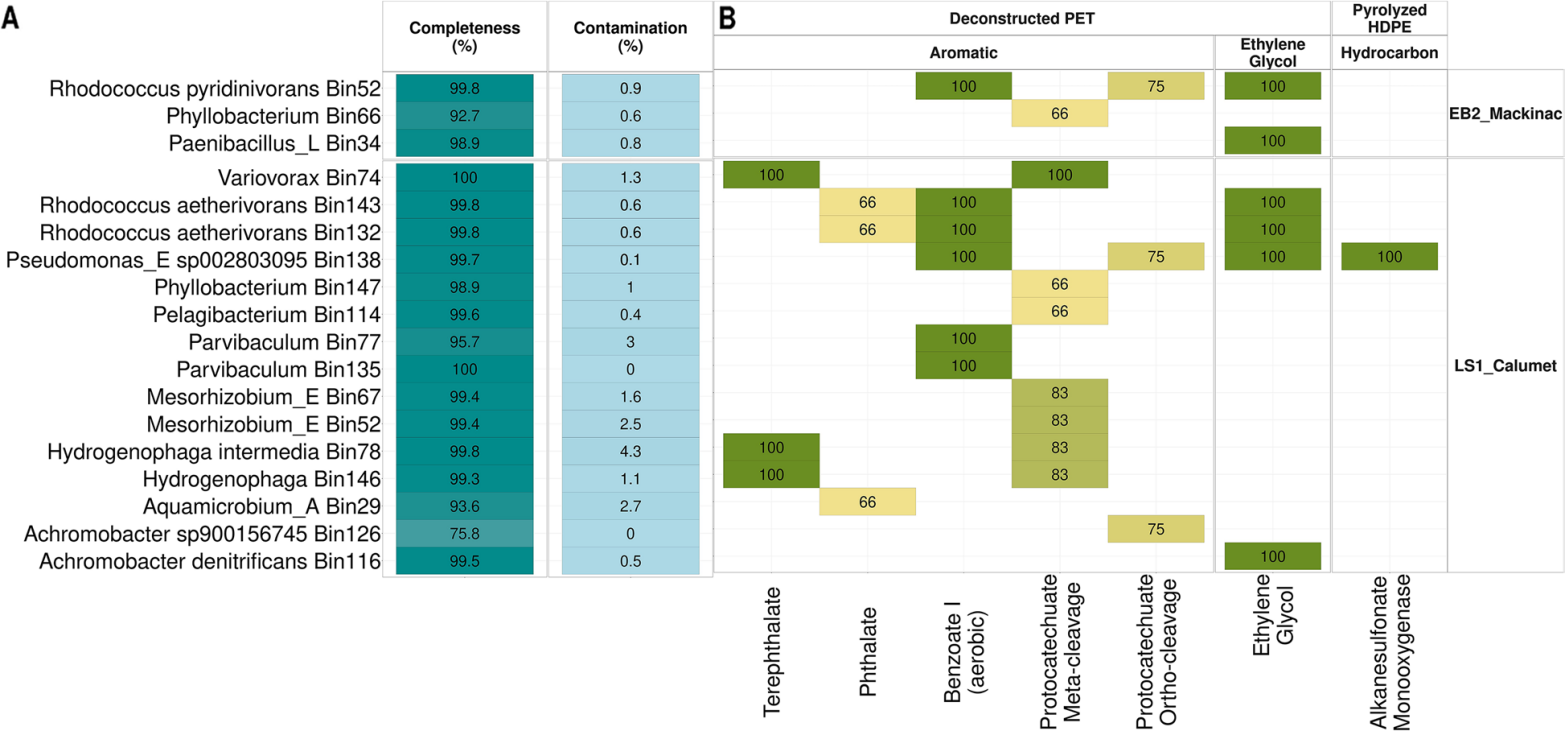
**A** Completeness and contamination of MAGs recovered from long-read metagenomic sequencing (Pacific Biosciences). **B** Percent completeness for relevant predicted pathways found in each MAG. Only MAGs greater than 70% complete are shown. Pathways greater than 80% complete were predicted to be functional. Only pathways greater than 50% complete are shown

Of the 12 MAGs obtained from the EB2_Mackinac enrichment, only three of them had relevant aromatic, ethylene glycol, and hydrocarbon degrading pathways. None of the MAGs from EB2_Mackinac had complete pathways for terephthalate or protocatechuate degradation (Fig. 4B), although partial pathways were present (Fig. S6). In the LS1_Calumet enrichment, three bins contained complete pathways for terephthalate degradation, and these bins were annotated as *Variovorax*, *Hydrogenophaga intermedia*, and *Hydrogenophaga* (unknown species) (Fig. 4B). Five bins in the LS1_Calumet enrichment were predicted to have functional pathways for degradation of protocatechuate: *Variovorax*, two *Mesorhizobium* bins, and the two *Hydrogenophaga* bins. In addition, partial protocatechuate pathways were present in several bins annotated as *Achromobacter*, *Phyllobacterium*, and *Pelagibacterium*.

### Pathways for biodegradation of deconstructed plastics

Interestingly, none of the *Rhodococcus* bins from either enrichment was predicted to have functional pathways for terephthalate and protocatechuate degradation, despite *Rhodococcus* being the most active genus when grown on terephthalate (Fig. 2). However, *Rhodococcus pyridinivorans* from the EB2_Mackinac enrichment and both of the *Rhodococcus aetherivorans* bins from the LS1_Calumet enrichment contained complete benzoate degradation pathways, which has been previously implicated in terephthalate degradation [50]. Three additional bins from the LS1_Calumet enrichment also contained the complete pathway for benzoate degradation including *Pseudomonas* and two *Parvibaculum* bins.

Although the microbial community sample collected for long-read metagenomic sequencing was grown on terephthalate, pathways were also found for ethylene glycol degradation as well as for hydrocarbon degradation, although we expect these pathways to be underrepresented in the long-read metagenomic sequencing data (Fig. 4B, Fig. S6). The only organism found to have a complete alkanesulfonate monooxygenase pathway (shown to degrade petroleum [51]) was the *Pseudomonas* bin from LS1_Calumet. Many organisms were found to have complete ethylene glycol degradation pathways including *Rhodococcus pyridinivorans* and *Paenibacillus* from the EB2_Mackinac enrichment, as well as *Pseudomonas* and both *Rhodococcus aetherivorans* bins from the LS1_Calumet enrichment.

## Discussion

In this study, we use natural microbial communities enriched to degrade polyethylene terephthalate and high-density polyethylene derivatives as a model system for investigating division of labor and the roles of specialist and generalist species in microbial communities. Much of the previous work using microorganisms for tandem chemical and biological plastic-upcycling has utilized isolates or bioengineered microorganisms to either convert waste PET into virgin materials or value-added compounds [10, 18, 52]. Other studies have shown the microbial communities are able to metabolize deconstructed plastics treated either with pyrolysis or chemical deconstruction [16, 53, 54]. Here, we use a microbial community instead of an isolate to demonstrate that a microbial community consists of specialist and generalist species, which allows flexibility to degrade processed mixed plastic waste. This finding has the potential to improve current recycling practices by allowing for processing of unsorted mixed plastic waste. Based on the community composition of the metatranscriptomic reads described here, *Rhodococcus* is present at > 2% relative abundance in all metatranscriptome communities (with the exception of the EB2_Mackinac ethylene glycol treatment), suggesting that *Rhodococcus* may be a generalist (Fig. 2). Based on analysis of bulk metatranscriptomic reads (Fig. 2), organisms from the genera *Rhodococcus* appear to be the most active organism in the deconstructed PET, terephthalate, and terephthalamide treatments for both enrichments, supporting the hypothesis that the communities would be dominated by generalist species. *Rhodococcus* MAGs recovered from short-read sequencing data from LS1_Calumet and EB2_Mackinac express phthalate, protocatechuate, and glycolate/glyoxylate genes in all treatments in both enrichments, suggesting that *Rhodococcus* is a generalist, able to grow on any of the substrates provided in this study (Fig. 3B). The *Rhodococcus* strains from both enrichments had reduced expression in the ethylene glycol treatment (Fig. 3B). One possible explanation for this is that these strains are negatively impacted by ethylene glycol, allowing some of the specialists in the community to compete more effectively in this treatment [44].

In the ethylene glycol treatments, there are high abundances of *Paracoccus* (EB2_Mackinac) and *Shinella* (LS1_Calumet), offering additional support for the presence of specialist organisms (Fig. 2). Based on bulk metatranscriptomic data, *Shinella* and *Paracoccus* are the primary ethylene glycol degraders in LS1_Calumet and EB2_Mackinac, respectively (Fig. 2). In the EB2_Mackinac enrichment, this is supported by the short-read metagenomic and metatranscriptomic analysis which shows higher gene expression from *Paracoccus* in the ethylene glycol and HDPE treatments (Fig. 3B). Unfortunately, the only *Shinella* MAG recovered from the LS1_Calumet enrichment was of very poor quality and so is not represented in these figures (contamination 94.9% and completeness 86.3%). The long-read metagenomic analysis presented here is limited because the sample collected was obtained from a culture grown on terephthalate, which limited our ability to obtain complete nearly single-contig MAGs for organisms preferring to grow on alternate substrates. Future long-read metagenomic sequencing of cultures grown on alternate substrates may allow for recovery of MAGs representing important specialists.

Additionally, the community composition of the HDPE metatranscriptome samples shows increased *Pseudomonas*, suggesting that members of the *Pseudomonas* may be possible HDPE specialists (Fig. 2). The *Pseudomonas* MAGs also were most active in the HDPE treatments (Fig. 3B) in both enrichments and were present only in very low abundances in other treatments, indicating that this may be another specialist (Fig. 2). *Rhodococcus* metatranscriptomic reads were present in all HDPE and ethylene glycol samples for both enrichments ranging from < 2 to 17.5% (lowest in the EB2_Mackinac ethylene glycol treatment) (Fig. 2). This suggests that *Rhodococcus* is still present in these treatments, even if it is abundant, supporting the theory that *Rhodococcus* is a generalist for the substrates.

Additional specialists were identified based on the short-read metagenomic and metatranscriptomic data, *Hydrogenophaga* from the LS1_Calumet enrichment is an aromatic-degrading specialist with the highest gene expression occurring in the aromatic treatments (deconstructed PET, terephthalate, and terephthalamide), having decreased or no expression in the ethylene glycol and HDPE treatments (Fig. 3B). *Paracoccus* in the LS1_Calumet enrichment only expresses the pathways of interest in the terephthalamide treatment, suggesting that this may be a terephthalamide specialist (Fig. 3B), unlike the *Paracoccus* in the EB2_Mackinac enrichment which primarily expresses genes in the ethylene glycol and HDPE treatments. Additionally, phthalate and benzoate-degrading genes were expressed in terephthalamide treatments, particularly by *Rhodococcus* (phthalate), and *Achromobacter* (benzoate) suggesting that these taxa may use these pathways for terephthalamide degradation as well (Fig. 3B). In both enrichments, *Pseudomonas* strains have the highest gene expression in the HDPE treatments (Fig. 3B). Based on gene expression, *Achromobacter*, *Hydrogenophaga*, *Paracoccus*, and *Pseudomonas* are examples of specialist organisms which have smaller niches compared to *Rhodococcus*. However, based on the long-read metagenomic data, *Pseudomonas* could also be defined as a generalist because it has the metabolic pathways for benzoate, ethylene glycol, and hydrocarbon degradation. This suggests that gene expression data may be important for defining the roles of specialists and generalists since metabolic pathways may be present but not be used. It is possible that these pathways are viable, but the organism is unable to grow because of competition with other organisms or other environmental constraints [44, 46].

In addition to the specialists and generalists described above, many low abundance organisms still have unclear roles in the community. Only incomplete phthalate pathways were identified in our MAGs (Fig. 4B) although relatively high expression of phthalate genes was noted in many of the short-read MAGs, particularly *Rhodococcus* and *Achromobacter*. In addition, *Hydrogenophaga* in the EB2_Mackinac enrichment and *Pseudomonas*, *Pelagibacterium*, and *Mesorhizobium* from the LS1_Calumet enrichment also expressed some phthalate genes (Fig. 3B). Previous work has demonstrated that the TCA cycle may be shared between taxa in complex relationships [46], which may be possible for other pathways as well.

Our secondary hypothesis for this study is that the terephthalate pathway will be shared between several members of the community. It has been well established that the terephthalate pathway converts terephthalate into protocatechuate which is degraded through one of three protocatechuate pathways [4]. Based on both short and long-read sequencing, *Hydrogenophaga* strains from both enrichments, and a *Variovorax* strain from LS1_Calumet are the only organisms which have both terephthalate and protocatechuate pathways (Figs. 3B and 4B). Our data show that *Hydrogenophaga* expresses both terephthalate and protocatechuate genes; however, there are additional organisms in the community, which lack the terephthalate pathway and express protocatechuate genes. These protocatechuate “specialists” include *Mesorhizobium* and *Pelagibacterium* (Figs. 3B and 4B). Many additional organisms lack any complete pathways for the degradation of the substrates used in this study, yet continue to persist in the community (Fig. S6). These observations taken together support that there may be division of labor in the enrichments described in this study. Data presented here suggests that metabolic division of labor is possible within the well-known terephthalate-degradation pathway. By using enriched microbial communities instead of isolates or synthetic microbial communities constructed from isolates, we expect to eliminate some of the biases associated with isolation techniques and preserve natural syntrophies between microorganisms [35].These observations could be confirmed in future studies where these organisms are isolated and co-cultured to confirm whether or not division of labor is involved in the terephthalate degradation pathway in these enrichments.

Interestingly, all of the *Rhodococcus* strains recovered from both EB2_Mackinac and LS1_Calumet lacked predicted functional terephthalate and protocatechuate pathways, despite being the most transcriptionally active. Furthermore, all MAGs recovered from the EB2_Mackinac consortium lacked pathways for terephthalate degradation (Fig. 4B). Alternate pathways for degradation of terephthalate have been previously reported in the literature: Choi et al. suggested that terephthalate is degraded through a bifurcated pathway involving phthalate genes [55]. The phthalate pathway converts phthalate to either protocatechuate or catechol, which are processed by central metabolisms [56–58]. In another study, terephthalate was also shown to be degraded through the benzoate pathway which yields succinyl-CoA and acetyl-CoA [50, 59]. Many peripheral enzymes exist to convert aromatic compounds into metabolites such as protocatechuate and catechol which are degraded by central cellular pathways; many of these enzymes have relaxed substrate specificity and may be able to degrade a wider range of compounds than what is currently known [60]. Complete benzoate pathways were identified in all three *Rhodococcus* strains identified in this study and in three strains from the LS1_Calumet enrichment: two *Parvibaculum* and one *Pseudomonas* (Fig. 4B). Expression of benzoate genes was noted in *Pelagibacterium* and *Achromobacter* MAGs from both enrichments as well as *Hydrogenophaga* from EB2_Mackinac and *Mesohrizobium* from LS1_Calumet (Fig. 3B). These observations support previous literature suggesting that the phthalate and benzoate pathways may have a role in degrading terephthalate.

## Conclusions

**Fig. 5:**
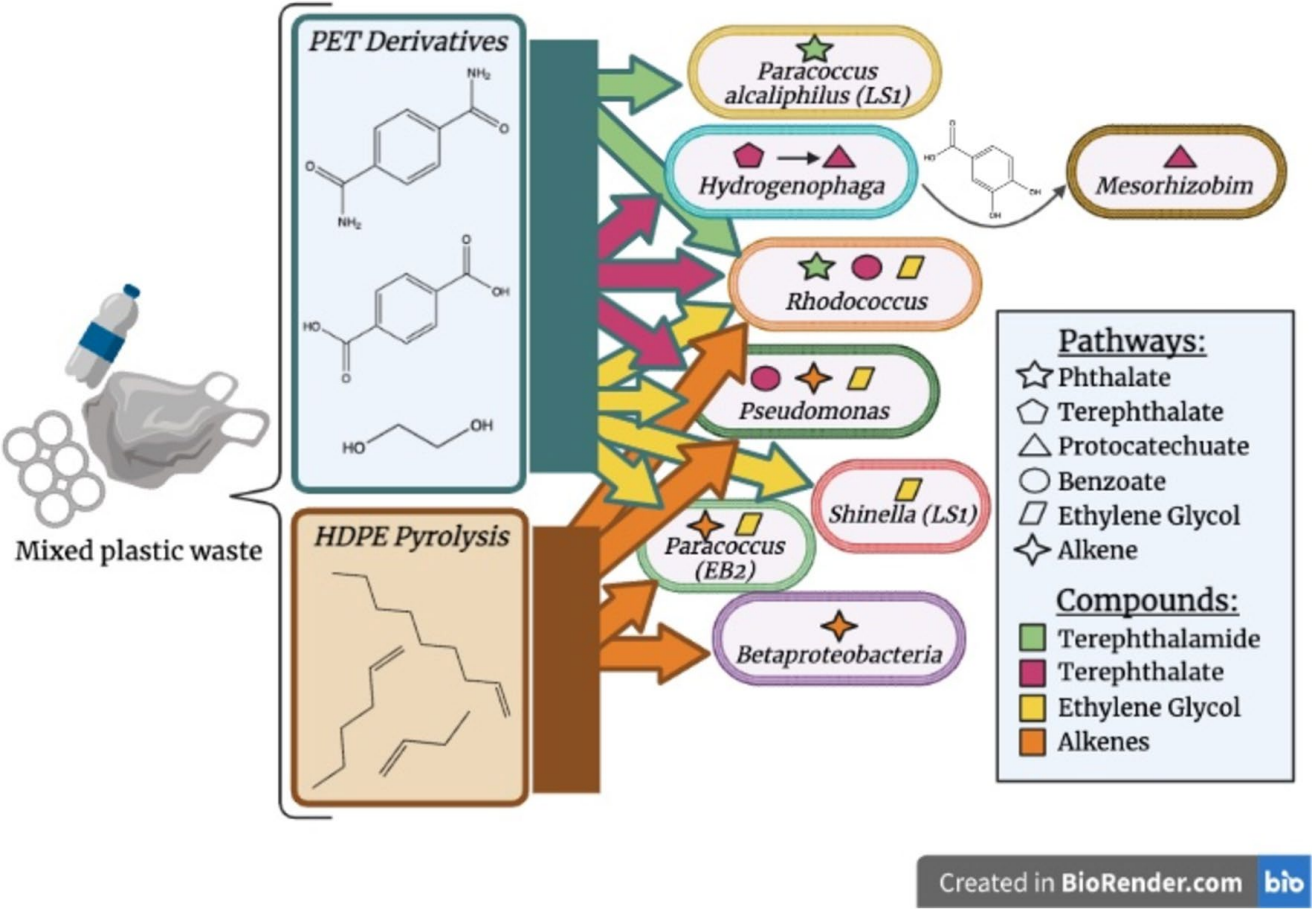
Overview of microbial community specialists, generalists, and division of labor

Data presented here shows that naturally enriched microbial communities can degrade mixed waste streams through division of labor by a community of both specialist and generalist microorganisms (Fig. 5). We demonstrated that specialist and generalist species within the community contribute to the community’s ability to process mixed plastic inputs and describe potential division of labor patterns in the communities. To gain a complete understanding of the community dynamics in these communities, future studies could look at gene expression and changes in abundance over time as these communities grow and degrade plastic derivatives. Additionally, metabolic modeling may be an invaluable tool to explore these communities in greater detail and under varying conditions. In silico experiments exploring pairwise interactions and leave-one-out wet lab experiments may inform the extent to which these organisms cooperate or compete. Flux balance analysis could also be used to optimize production of biomass or other metabolites for future industrial applications.

## Methods

### HDPE pyrolysis process

HDPE was converted into pyrolysis oil using a novel liquid-fed pyrolysis tubular reactor at 575 °C and with a vapor residence time of 1 s. Experimental methods for the liquid-feed pyrolysis system have been previously described by Byrne et al. [53] and Kulas et al. [61]. The collected pyrolysis oil product was batch distilled to remove the heavier components (> C15), creating a pyrolysis oil consisting of C6-C15 alkene hydrocarbons. Experimental methods for the batch distillation and the distribution of hydrocarbon compounds in this range have been previously described by Szwaja et al. [62].

### PET chemical deconstruction process

PET plastic cups were size reduced using a knife mill (Crumbler®, Forest Concepts, Auburn, WA) to 30 mm, then further size reduced to 3–5 mm using the Crumbler®. The milled PET was added to a custom extraction vessel and placed under −10 psig vacuum. Liquid DME was added to the extraction chamber, submerging the solids, and held at room temperature for 20 min before being transferred to a second chamber. The cleaned plastic was removed and dried at room temperature. The PET cup particles were then processed in a custom horizontal stainless steel reactor (550 mL capacity) using 10 wt% NH_4_OH, at 0.25 g PET/mL NH_4_OH solids loading, at 240 °C for 60-min residence time following heating. PET was added manually to the reactor followed by manual addition of ammonium hydroxide. At the end of the residence time, the vessel was cooled and the liquid recovered and vacuum-filtered through Whatman #42 filter paper (diameter 55 mm, pore size 2.5 μm). The solids were dried at 55 °C and used to determine solubilization. The liquid was pH-adjusted from 10.4 to 7 using phosphoric acid. The final liquid product was filtered through a 0.2-μm polyethersulfone filter before being added to the microbial culture.

### Culture growth

LS1_Calumet and EB2_Mackinac consortia have been previously described by Schaerer et al. [54] and Byrne et al. [53]. The LS1_Calumet enrichment was enriched from a compost sample collected from a farm in Calumet, MI (Coordinates 47.211, − 88.553) by adding 1 g of compost per 100 mL of 10 g/L disodium terephthalate in Bushnell Haas medium, incubating at room temperature stirring continuously. Every 14 days the culture was transferred to fresh Bushnell Haas medium amended with 10 g/L disodium terephthalate with 10% inoculum for a total of four transfers after which it was maintained by weekly replacing 40% of the volume with fresh Bushnell Haas medium amended with disodium terephthalate. The EB2_Mackinac culture was enriched from sediment collected from the Straits of Mackinac (Coordinates 46.532, −88.141) and the same compost sample as the LS1_Calumet enrichment. The culture was started by adding 2 g of inoculum from each of the two sources into 100 mL of Bushnell Haas medium and amending the culture with 125 µL of pyrolysis-treated polypropylene products (previously described by Byrne et al. [53]). The culture was incubated at room temperature, stirring continuously. The culture was transferred daily for 5 days, then maintained the same as the LS1_Calumet culture described above.

In the present experiment, EB2_Mackinac and LS1_Calumet were each grown on five substrates (chemically deconstructed PET, ethylene glycol, disodium terephthalate, solubilized terephthalamide, and HDPE pyrolysis product). Cultures were grown at 10 g/L of each substrate. Terephthalamide was solubilized using hydrochloric acid and neutralized with sodium hydroxide. Cultures were grown at 50 mL volume and incubated at 37 °C while mixing on stir plates with teflon-coated magnetic stir bars at 130 rpm. Cultures were incubated for 136 h (~6 days). Optical density (OD_600_) was used to measure growth during the incubation. Daily 1 mL subsamples were collected and placed in plastic cuvettes. OD_600_ was measured using a Genesys 10S UV-VIS Spectrophotometer (Thermo Scientific). Growth data was imported into R and was plotted using ggplot2 [63, 64].

### Nucleic acid extraction and sequencing

At the end of the experiment, two 10 mL subsamples were collected from each culture, centrifuged at 10,000 × g for 10 min, and decanted. The resulting cell pellets were used for DNA and RNA extractions using MP Biomedicals extraction kits according to the manufacturer instructions. Illumina metagenomic sequencing was performed at the University of Utah Huntsman Cancer Institute High-throughput Genomics Facility. Metagenomics libraries were produced from extracted DNA using the Illumina Nextera DNA Flex Library Prep. Libraries were sequenced on the Illumina NovaSeq using a paired end 2 × 150 bp kit. Metatranscriptomic sequencing was performed at the Microbial Genome Sequencing Center (MiGS). Metatranscriptome libraries were prepared using Illumina’s RiboZero Plus kit. Sequencing was performed on the Illumina NextSeq 2000 sequencer using a paired end 2 × 50 bp kit. Additionally, to confirm the presence and absence of genes and pathways in each taxon, long-read metagenomic sequencing was performed to obtain higher quality genomes. PacBio HiFi sequencing was performed at Maryland Genomics. The cultures for long-read metagenomic sequencing were grown on 10 g/L disodium terephthalate in Bushnell Haas medium as described above. DNA was extracted using the MP Biomedicals FastSoil DNA kit. R [63] was used to calculate statistics and create figures from all data collected in this study; our analysis pipelines are publicly available at: https://github.com/lgschaer/MiniOmics.

### Community composition

Kraken 2 [65] was used to assign taxonomy to metagenomic and metatranscriptomic reads. Cleaned Illumina readers were processed through kraken2 and classified using the Standard-8 database. Bracken [66] was used to calculate abundances from the kraken2 output. Abundance percentages obtained from Kraken were imported into R [63, 64] to estimate microbial community composition using phyloseq [67]. The “stats” package was used to perform a Kruskal-Wallis test to determine whether there was a difference in alpha diversity between the metagenomic and metatranscriptomic reads. The “vegan” package was used to perform a PERMANOVA to test for significant differences in community composition between treatments [68].

### Short-read metagenomics and metatranscriptomics workflow

Raw Illumina metagenomic sequencing files were concatenated to obtain a single file for the forward reads and a single file for the reverse reads for each of the two consortia. Forward and reverse reads were interleaved using BBMap [69] to create a single fastq file for each of the consortia. Reads were quality-filtered based on the default parameters and then assembled using MEGAHIT [70]. Assembled reads were indexed and mapped using Bowtie2 [71] and SAMtools [72]. Reads were separated into bins using MetaBat2 [73]. Completeness and contamination were evaluated with CheckM [74]. Bins were grouped into quality categories based on the following criteria: high (completeness > 90%, contamination < 5%), medium (completeness ≥ 50%, contamination < 10%), low (completeness < 50%, contamination < 10%), and contaminated (contamination ≥ 10%). Taxonomy was assigned to each bin using BAT which uses the NCBI non-redundant protein database, which is assembled and searched with DIAMOND [75, 76], and genes were annotated using Prokka [77]. Relevant metabolic pathways were identified by running gapseq on the assembled contigs [78]. Results were further analyzed in R [63, 64].

First raw Illumina metatranscriptomic reads were quality-trimmed using Trimmomatic (adapter sequences removed, leading and trailing ends were trimmed if below a quality score of 3, sliding window was set to 4:15, reads below 36 bp were dropped from the data set) [79]. Reference genomes were constructed from the metagenome assembly fasta files using Bowtie2 [71]. Reads were then mapped to the reference database using Bowtie2 [71]. Counts were obtained with HTSeq [80]. Results were then imported into R for further analysis [63, 64].

Expressed genes were categorized into pathways known to process the compounds of interest, which were previously identified using gapseq. The log of the sum of expressed gene counts for each pathway is shown in Fig. 3B; pathway completeness is not considered for the short-read metagenomic and transcriptomic sequencing results. Briefly, the polyethylene terephthalate pathway feeds into the terephthalate pathway [81], which feeds into one of three protocatechuate pathways (meta [82], para [83], or ortho-cleavage [84]). The protocatechuate pathways yield compounds which are processed through central cellular metabolisms, either the β-ketoadipate pathway or the TCA cycle [82–84]. Alternatively, the phthalate pathway has been shown to be involved in terephthalate biodegradation yielding protocatechuate or catechol [85]. The benzoate pathway, which yields catechol, was also previously shown to process terephthalate [50, 86]. Ethylene glycol is processed through the glycolate and glyoxylate pathways which yields 2-phospho-d-glycerate [87, 88]. The full metabolic maps have been previously described by others [4, 17]. The alkanesulfonate pathway has been previously implicated in hydrocarbon degradation [51].

### Long-read metagenomics workflow

To determine which taxa had complete pathways for degradation of the various plastic derivative substrates and to be confident that certain taxa did not have certain genes and pathways, long-read metagenomic sequencing was performed to obtain higher quality MAGs. Raw PacBio metagenomic reads were assembled using hifiasm-meta [89]. After assembly, reads were processed according to the Pacific Biosciences (PacBio) HiFi MAG Pipeline, a snakemake workflow. Briefly, the PacBio workflow used Minimap2 [90] to map the reads to the contigs, MetaBat2 [73] to bin contigs, CheckM [74] to assess quality, and GTDB-Tk [91] to assign taxonomy. We also assigned taxonomy to our bins using BAT [75] to compare annotations with GTDB-Tk and annotated genes with Prokka [77]. Gapseq was used to estimate completeness of relevant metabolic pathways [78].

### Supplementary Information

**Additional file 1: Table S1.** Composition of the chemically deconstructed PET. **Table S2.** Maximum change in growth (OD_600_) for each enrichment on each substrate relative to the uninoculated blanks. DPCET (chemically deconstructed PET), TPA (terephthalate), TA (terephthalamide), EG (ethylene glycol), HDPE (pyrolyzed high density polyethylene). **Figure S1.** Observed alpha diversity of metagenome and metatranscriptome samples. Sequence type abbreviations: MT (metatranscriptomic) or MG (metagenomic). Substrate abbreviations: high density polyethylene pyrolysis (HDPE), deconstructed PET (DCPET), terephthalate (TPA), terephthalamide (TA), and ethylene glycol (EG). **Figure S2.** Shannon alpha diversity of metagenome and metatranscriptome samples. Sequence type abbreviations: MT (metatranscriptomic) or MG (metagenomic). Substrate abbreviations: high density polyethylene pyrolysis (HDPE), deconstructed PET (DCPET), terephthalate (TPA), terephthalamide (TA), and ethylene glycol (EG). **Table S3.** Shannon and Observed alpha diversity of metagenomic (MG) and metatranscriptomic (MT) samples. Substrate abbreviations: high density polyethylene pyrolysis (HDPE), deconstructed PET (DCPET), terephthalate (TPA), terephthalamide (TA), and ethylene glycol (EG). **Table S4.** Kruskal-Wallis comparison of alpha diversity between sequence types (metagenome versus metatranscriptome). **Figure S3.** Bray Curtis principal coordinates analysis (PCoA). Shapes denote sequence type: MT (metatranscriptomic) or MG (metagenomic). Substrate abbreviations: high density polyethylene pyrolysis (HDPE), deconstructed PET (DCPET), terephthalate (TPA), terephthalamide (TA), and ethylene glycol (EG). **Table S5.** Results summary of PERMANOVA comparison of microbial community composition between sequence type (metagenomic versus metatranscriptomic samples). **Table S6.** Results summary of PERMANOVA comparison of microbial community composition between aromatic metatranscriptomic samples (deconstructed PET, terephthalate, and terephthalamide) and non-aromatic metatranscriptomic samples (HDPE pyrolysis and ethylene glycol). **Table S7.** Table summary of the percentage of metagenomic (MG) and metatranscriptomic (MT) reads assigned to each genus from each treatment. The numbers represent the percentage of total reads in each sample belonging to each genus. Percentages may not add up to 100% due to rounding. **Figure S4.** Comparison of the completeness, contamination, and number of contigs in Illumina and PacBio MAGs (medium and high quality only). EB2 (EB2_Mackinac), LS1 (LS1_Calumet). Note changing scales on the y-axis. **Table S8.** Summary of completeness, contamination and number of contigs in short-read (Illumina) and long-read (Pacific Biosciences) MAGs. **Figure S5.** (A) Completeness and contamination of MAGs obtained from short-read metagenomic sequencing. MAGs with contamination greater than 10% are not shown. (B) Gene expression of genes in relevant pathways in each treatment. Numbers shown are the log10 of the sum of htseq counts of genes in each pathway. **Figure S6.** Relevant predicted pathways for all MAGs recovered from long-read metagenomic sequencing (Pacific Biosciences). Pathways greater than 80% complete are expected to be functional. **Table S9.** Summary of high and medium quality MAGs recovered from long-read metagenomic sequencing. **Table S10.** Summary of high and medium quality MAGs recovered from short-read metagenomic sequencing.

## Data Availability

The raw sequencing reads have been deposited in the SRA under bioproject PRJNA947631.

## References

[1] Geyer R (2017). Production, use, and fate of all plastics ever made. Sci Adv.

[2] Vollmer I (2020). Beyond mechanical recycling: giving new life to plastic waste. Angew Chem Int Ed.

[3] Schaerer LG (2022). Killing two birds with one stone: chemical and biological upcycling of polyethylene terephthalate plastics into food. Trends Biotechnol.

[4] Dissanayake L, Jayakody LN (2021). Engineering microbes to bio-upcycle polyethylene terephthalate. Front Bioeng Biotechnol.

[5] Kenny S (2012). Development of a bioprocess to convert PET derived terephthalic acid and biodiesel derived glycerol to medium chain length polyhydroxyalkanoate. Appl Microbiol Biotechnol.

[6] Liu P (2021). Potential one-step strategy for PET degradation and PHB biosynthesis through co-cultivation of two engineered microorganisms. Eng Microbiol.

[7] Guzik MW (2021). Robust process for high yield conversion of non-degradable polyethylene to a biodegradable plastic using a chemo-biotechnological approach. Waste Manage.

[8] Guzik M (2014). Conversion of post consumer polyethylene to the biodegradable polymer polyhydroxyalkanoate. Appl Microbiol Biotechnol.

[9] Werner AZ (2021). Tandem chemical deconstruction and biological upcycling of poly(ethylene terephthalate) to β-ketoadipic acid by Pseudomonas putida KT2440. Metab Eng.

[10] Sullivan KP (2022). Mixed plastics waste valorization through tandem chemical oxidation and biological funneling. Science.

[11] Nakkabi A (2015). Biological degradation of polyurethane by a newly isolated wood bacterium. Int J Recent Adv Multidiscip Res..

[12] Auta H (2018). Growth kinetics and biodeterioration of polypropylene microplastics by Bacillus sp. and Rhodococcus sp. isolated from mangrove sediment. Mar Pollut Bull.

[13] Chang Y-C (2015). Whole-genome sequence of Aquamicrobium sp. strain SK-2, a polychlorinated biphenyl-utilizing bacterium isolated from sewage sludge. Genome Announc (Washington, DC).

[14] Meyer-Cifuentes I (2020). Synergistic biodegradation of aromatic-aliphatic copolyester plastic by a marine microbial consortium. Nat Commun.

[15] Roberts C (2020). Environmental consortium containing pseudomonas and bacillus species synergistically degrades polyethylene terephthalate plastic. mSphere.

[16] Putman LI, Laura GS, Wu R, Kulas DG, Ali Z, Ong RG, et al. Deconstructed Plastic Substrate Preferences of Microbial Populations from the Natural Environment. Microbiol Spectr. 2023;e00362–23. 10.1128/spectrum.00362-23 PMC10433879 37260392

[17] Qi X (2021). Current advances in the biodegradation and bioconversion of polyethylene terephthalate. Microorganisms.

[18] Sadler JC, Wallace S (2021). Microbial synthesis of vanillin from waste poly(ethylene terephthalate). Green Chem.

[19] Lee Y (2019). Biodegradation of naphthalene, BTEX, and aliphatic hydrocarbons by Paraburkholderia aromaticivorans BN5 isolated from petroleum-contaminated soil. Sci Rep.

[20] Sarmah P, Rout J (2018). Efficient biodegradation of low-density polyethylene by cyanobacteria isolated from submerged polyethylene surface in domestic sewage water. Environ Sci Pollut Res.

[21] Yoshida S (2021). Ideonella sakaiensis, PETase, and MHETase: from identification of microbial PET degradation to enzyme characterization. Methods Enzymol.

[22] Chamas A (2020). Degradation rates of plastics in the environment. ACS Sustain Chem Eng.

[23] Gravel D (2011). Experimental niche evolution alters the strength of the diversity–productivity relationship. Nature.

[24] Fischer K, Majewsky M (2014). Cometabolic degradation of organic wastewater micropollutants by activated sludge and sludge-inherent microorganisms. Appl Microbiol Biotechnol.

[25] Shangpliang H (2018). Bacterial community in naturally fermented milk products of Arunachal Pradesh and Sikkim of India analysed by high-throughput amplicon sequencing. Sci Rep.

[26] Einson JE (2018). A vegetable fermentation facility hosts distinct microbiomes reflecting the production environment. Appl Environ Microbiol.

[27] Mariadassou M (2015). Microbial ecosystems are dominated by specialist taxa. Ecol Lett.

[28] Julliard R (2006). Spatial segregation of specialists and generalists in bird communities. Ecol Lett.

[29] Rombouts JL (2019). The impact of mixtures of xylose and glucose on the microbial diversity and fermentative metabolism of sequencing-batch or continuous enrichment cultures. FEMS Microbiol Ecol.

[30] Futuyma DJ, Moreno G (1988). The evolution of ecological specialization. Annu Rev Ecol Syst.

[31] Xu Q (2022). Microbial generalists and specialists differently contribute to the community diversity in farmland soils. J Adv Res.

[32] Székely AJ, Langenheder S (2014). The importance of species sorting differs between habitat generalists and specialists in bacterial communities. FEMS Microbiol Ecol.

[33] Shink B (2002). Synergistic interactions in the microbial world. Antonie Van Leeuwenhoek.

[34] Palmer JD, Foster KR (2022). Bacterial species rarely work together. Science.

[35] Foster KR, Bell T (2012). Competition, not cooperation, dominates interactions among culturable microbial species. Curr Biol.

[36] Coyte KZ (2015). The ecology of the microbiome: networks, competition, and stability. Science.

[37] Morris BEL (2013). Microbial syntrophy: interaction for the common good. FEMS Microbiol Rev.

[38] West SA (2007). Evolutionary explanations for cooperation. Curr Biol.

[39] Tsoi R (2019). Emerging strategies for engineering microbial communities. Biotechnol Adv.

[40] Borchert E (2021). Enhancing microbial pollutant degradation by integrating eco-evolutionary principles with environmental biotechnology. Trends Microbiol.

[41] Tucci M (2022). Syntrophy drives the microbial electrochemical oxidation of toluene in a continuous-flow “bioelectric well”. J Environ Chem Eng.

[42] Qi M (2021). Microbial interactions drive the complete catabolism of the antibiotic sulfamethoxazole in activated sludge microbiomes. Environ Sci Technol.

[43] Zengler K, Zaramela LS (2018). The social network of microorganisms — how auxotrophies shape complex communities. Nat Rev Microbiol.

[44] Wang M (2022). Substrate availability and toxicity shape the structure of microbial communities engaged in metabolic division of labor. mLife.

[45] Tsoi R (2018). Metabolic division of labor in microbial systems. Proc Natl Acad Sci USA.

[46] Thommes M (2019). Designing metabolic division of labor in microbial communities. mSystems.

[47] Bao T, Qian Y, Xin Y, Collins JJ, Lu T (2023). Engineering microbial division of labor for plastic upcycling. Nat Commun..

[48] Ragaert K (2017). Mechanical and chemical recycling of solid plastic waste. Waste Manage.

[49] Chaudhari US (2021). Systems analysis approach to polyethylene terephthalate and olefin plastics supply chains in the circular economy: a review of data sets and models. ACS Sustain Chem Eng.

[50] Kleerebezem R (1999). The role of benzoate in anaerobic degradation of terephthalate. Appl Environ Microbiol.

[51] Li S-W (2020). Transcriptome profiling reveals the molecular processes for survival of Lysinibacillus fusiformis strain 15–4 in petroleum environments. Ecotoxicol Environ Saf.

[52] Lu H (2022). Machine learning-aided engineering of hydrolases for PET depolymerization. Nature.

[53] Byrne E (2022). Pyrolysis-aided microbial biodegradation of high-density polyethylene plastic by environmental inocula enrichment cultures. ACS Sustainable Chem Eng.

[54] Schaerer LG, Emily W, Sulihat A, Emily BM, Aamir Br, Kaushik B, et al. Versatile microbial communities rapidly assimilate ammonium hydroxide-treated plastic waste. J Ind Microbiol Biotechnol. 2023;50(1):kuad008. 10.1093/jimb/kuad008 PMC10124128 37061790

[55] Choi KY (2005). Molecular and biochemical analysis of phthalate and terephthalate degradation by *Rhodococcus* sp. strain DK17. FEMS Microbiol Lett.

[56] Martínková L (2009). Biodegradation potential of the genus Rhodococcus. Environ Int.

[57] Pérez-Pantoja D, González B, Pieper DH, Timmis KN (2010). Aerobic Degradation of Aromatic Hydrocarbons. Handbook of Hydrocarbon and Lipid Microbiology.

[58] Patrauchan MA (2005). Catabolism of benzoate and phthalate in *Rhodococcus* sp. strain RHA1: redundancies and convergence. J Bacteriol.

[59] Valderrama JA (2012). Bacterial degradation of benzoate. J Biol Chem.

[60] Pérez-Pantoja D (2008). Metabolic reconstruction of aromatic compounds degradation from the genome of the amazing pollutant-degrading bacterium *Cupriavidus necator* JMP134. FEMS Microbiol Rev.

[61] Kulas DG (2022). Liquid-fed waste plastic pyrolysis pilot plant: effect of reactor volume on product yields. J Anal Appl Pyrol.

[62] Szwaja M (2022). Comparative analysis of injection of pyrolysis oil from plastics and gasoline into the engine cylinder and atomization by a direct high-pressure injector. Energies.

[63] R Core Team. R: a language and environment for statistical computing. 2013.

[64] Wickham H (2019). Welcome to the Tidyverse. JOSS.

[65] Wood DE (2019). Improved metagenomic analysis with Kraken 2. Genome Biol.

[66] Lu J (2017). Bracken: estimating species abundance in metagenomics data. PeerJ Comput Sci.

[67] McMurdie PJ, Holmes S (2013). Phyloseq: an R package for reproducible interactive analysis and graphics of microbiome census data. PLoS One.

[68] Dixon P (2003). VEGAN, a package of R functions for community ecology. J Veg Sci.

[69] Bushnell B. BBMap: a fast, accurate, splice-aware aligner. 2014.

[70] Li D (2015). MEGAHIT: an ultra-fast single-node solution for large and complex metagenomics assembly via succinct de Bruijn graph. Bioinformatics (Oxford, England).

[71] Langdon WB (2015). Performance of genetic programming optimised Bowtie2 on genome comparison and analytic testing (GCAT) benchmarks. BioData Min.

[72] Danecek P (2021). Twelve years of SAMtools and BCFtools. GigaScience.

[73] Kang DD (2019). MetaBAT 2: an adaptive binning algorithm for robust and efficient genome reconstruction from metagenome assemblies. PeerJ.

[74] Parks DH (2015). CheckM: assessing the quality of microbial genomes recovered from isolates, single cells, and metagenomes. Genome Res.

[75] von Meijenfeldt FAB (2019). Robust taxonomic classification of uncharted microbial sequences and bins with CAT and BAT. Genome Biol.

[76] Buchfink B (2015). Fast and sensitive protein alignment using DIAMOND. Nat Methods.

[77] Seemann T (2014). Prokka: rapid prokaryotic genome annotation. Bioinformatics (Oxford, England).

[78] Zimmermann J (2021). gapseq: informed prediction of bacterial metabolic pathways and reconstruction of accurate metabolic models. Genome Biol.

[79] Bolger AM (2014). Trimmomatic: a flexible trimmer for Illumina sequence data. Bioinformatics.

[80] Anders S (2015). HTSeq–a Python framework to work with high-throughput sequencing data. Bioinformatics.

[81] Yoshida S (2016). A bacterium that degrades and assimilates poly(ethylene terephthalate). Science (New York, N.Y.).

[82] Kamimura N, Masai E. The protocatechuate 4,5-cleavage pathway: overview and new findings. In: Nojiri H, et al., editors. Biodegradative bacteria. Springer Japan; 2014. pp. 207–226.

[83] Kasai D (2009). Uncovering the protocatechuate 2,3-cleavage pathway genes. J Bacteriol.

[84] Okamura-Abe Y (2016). Beta-ketoadipic acid and muconolactone production from a lignin-related aromatic compound through the protocatechuate 3,4-metabolic pathway. J Biosci Bioeng.

[85] Hara H (2007). Transcriptomic analysis reveals a bifurcated terephthalate degradation pathway in *Rhodococcus* sp. strain RHA1. J Bacteriol.

[86] Altenschmidt U (1993). New aerobic benzoate oxidation pathway via benzoyl-coenzyme A and 3-hydroxybenzoyl-coenzyme A in a denitrifying Pseudomonas sp. J Bacteriol.

[87] Mückschel B (2012). Ethylene glycol metabolism by Pseudomonas putida. Appl Environ Microbiol.

[88] Trifunović D (2016). Ethylene glycol metabolism in the acetogen Acetobacterium woodii. J Bacteriol.

[89] Feng X (2022). Metagenome assembly of high-fidelity long reads with hifiasm-meta. Nat Methods.

[90] Li H (2018). Minimap2: pairwise alignment for nucleotide sequences. Bioinformatics.

[91] Chaumeil P-A (2019). GTDB-Tk: a toolkit to classify genomes with the Genome Taxonomy Database. Bioinformatics.

