# Supplementary material for: Coexistence of specialist and generalist species within mixed plastic derivative-utilizing microbial communities
Source: Microbiome. 2023 Oct 14;11:224. doi: 10.1186/s40168-023-01645-4 (PMC10576394; doi:10.1186/s40168-023-01645-4)

**Supplemental Materials: Coexistence of specialist and generalist species within mixed plastic derivative-utilizing microbial communities**

Laura Schaerer^1^, Lindsay Putman^1^, Isaac Bigcraft^1^, Emma Byrne^1^, Daniel Kulas^2^, Ali Zolghadr^2^, Sulihat Aloba^2^, Rebecca Ong^2^, David Shonnard^2^, Stephen Techtmann^1*^

^1^Department of Biological Sciences Michigan Technological University, Houghton MI USA

^2^Department of Chemical Engineering Michigan Technological University, Houghton MI USA

*Corresponding author: Stephen Techtmann

740 Dow ESE building

1400 Townsend Drive

Houghton, MI 49931

[smtechtm@mtu.edu](mailto:smtechtm@mtu.edu)

Keyword: Microbial community, plastic upcycling, specialist, generalist, biotechnology

**Table S1** Composition of the chemically deconstructed PET.

| ***Compound*** | ***Concentration (g/L)*** |
| --- | --- |
| Terephthalic Acid | 299 |
| Terephthalic Acid Monoamide | 150 |
| Terephthalamide | Not detected |
| Ethylene Glycol | 71 |

**Table S2**. Maximum change in growth (OD_600_) for each enrichment on each substrate relative to the uninoculated blanks. DPCET (chemically deconstructed PET), TPA (terephthalate), TA (terephthalamide), EG (ethylene glycol), HDPE (pyrolyzed high density polyethylene).

| ***Community*** | ***DCPET*** | ***TPA*** | ***TA*** | ***EG*** | ***HDPE*** |
| --- | --- | --- | --- | --- | --- |
| EB2_Mackinac | 1.139 | 0.474 | 1.576 | 0.129 | 0.535 |
| LS1_Calumet | 0.842 | 0.331 | 1.378 | 0.134 | 0.367 |
| Uninoculated Blank | 0.003 | 0.005 | 0.072 | 0.014 | 0.114 |


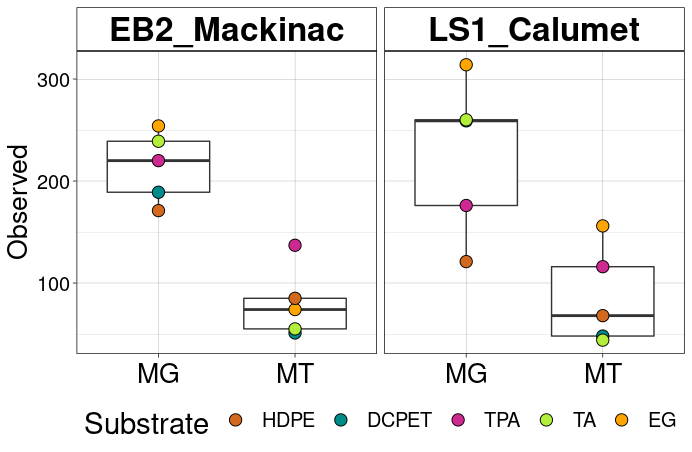


**Figure S1**. Observed alpha diversity of metagenome and metatranscriptome samples. Sequence type abbreviations: MT (metatranscriptomic) or MG (metagenomic). Substrate abbreviations: high density polyethylene pyrolysis (HDPE), deconstructed PET (DCPET), terephthalate (TPA), terephthalamide (TA), and ethylene glycol (EG).


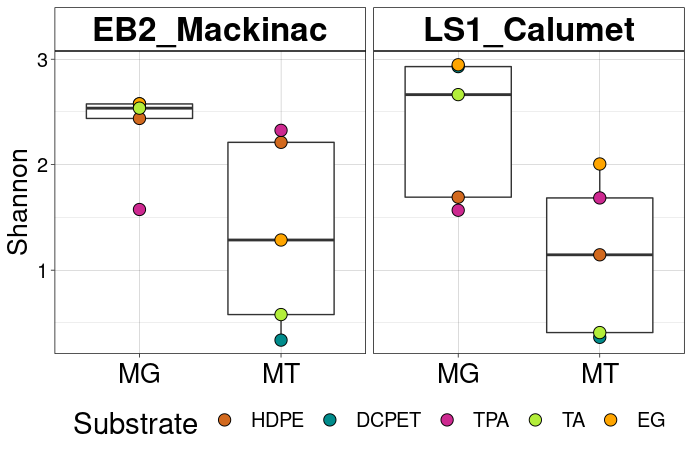


**Figure S2**. Shannon alpha diversity of metagenome and metatranscriptome samples. Sequence type abbreviations: MT (metatranscriptomic) or MG (metagenomic). Substrate abbreviations: high density polyethylene pyrolysis (HDPE), deconstructed PET (DCPET), terephthalate (TPA), terephthalamide (TA), and ethylene glycol (EG).

**Table S3**. Shannon and Observed alpha diversity of metagenomic (MG) and metatranscriptomic (MT) samples. Substrate abbreviations: high density polyethylene pyrolysis (HDPE), deconstructed PET (DCPET), terephthalate (TPA), terephthalamide (TA), and ethylene glycol (EG).

| ***Enrichment*** | ***Substrate*** | ***Observed*** | | ***Shannon*** | |
| --- | --- | --- | --- | --- | --- |
|  |  | ***MG*** | ***MT*** | ***MG*** | ***MT*** |
| EB2 Mackinac | HPDE | 171 | 85 | 2.44 | 2.21 |
|  | DCPET | 189 | 51 | 2.58 | 0.33 |
|  | TPA | 220 | 137 | 1.58 | 2.33 |
|  | TA | 239 | 55 | 2.54 | 0.58 |
|  | EG | 254 | 74 | 2.58 | 1.29 |
| LS1 Calumet | HPDE | 121 | 68 | 1.69 | 1.15 |
|  | DCPET | 259 | 48 | 2.93 | 0.36 |
|  | TPA | 176 | 116 | 1.57 | 1.68 |
|  | TA | 262 | 44 | 2.66 | 0.41 |
|  | EG | 314 | 156 | 2.95 | 2.01 |

**Table S4**. Kruskal-Wallis comparison of alpha diversity between sequence types (metagenome versus metatranscriptome).

| ***Metric*** | ***Chi-Squared*** | ***Degrees of Freedom*** | ***P-Value*** |
| --- | --- | --- | --- |
| Observed | 13.166 | 1 | 0.0002851 |
| Shannon | 8.6914 | 1 | 0.003197 |


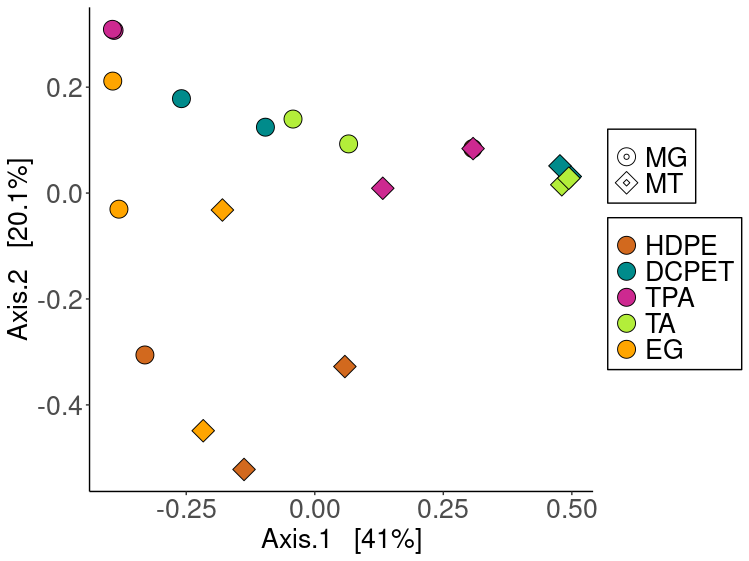


**Figure S3**. Bray Curtis principal coordinates analysis (PCoA). Shapes denote sequence type: MT (metatranscriptomic) or MG (metagenomic). Substrate abbreviations: high density polyethylene pyrolysis (HDPE), deconstructed PET (DCPET), terephthalate (TPA), terephthalamide (TA), and ethylene glycol (EG).

**Table S5**. Results summary of PERMANOVA comparison of microbial community composition between sequence type (metagenomic versus metatranscriptomic samples).

| ***Comparison*** | ***Degrees of Freedom*** | ***Sum of Squares*** | ***R^2^*** | ***F-Statistic*** | ***P-Value*** |
| --- | --- | --- | --- | --- | --- |
| Metagenomic vs. Metatranscriptomic | 1 | 1.0601 | 0.21 | 4.8311 | 0.003 |

**Table S6**. Results summary of PERMANOVA comparison of microbial community composition between aromatic metatranscriptomic samples (deconstructed PET, terephthalate, and terephthalamide) and non-aromatic metatranscriptomic samples (HDPE pyrolysis and ethylene glycol).

| ***Comparison*** | ***Degrees of Freedom*** | ***Sum of Squares*** | ***R^2^*** | ***F-Statistic*** | ***P-Value*** |
| --- | --- | --- | --- | --- | --- |
| Aromatic vs. Non-aromatic | 1 | 1.0114 | 0.44 | 6.2623 | 0.006 |

**Table S7**. Table summary of the percentage of metagenomic (MG) and metatranscriptomic (MT) reads assigned to each genus from each treatment. The numbers represent the percentage of total reads in each sample belonging to each genus. Percentages may not add up to 100% due to rounding.


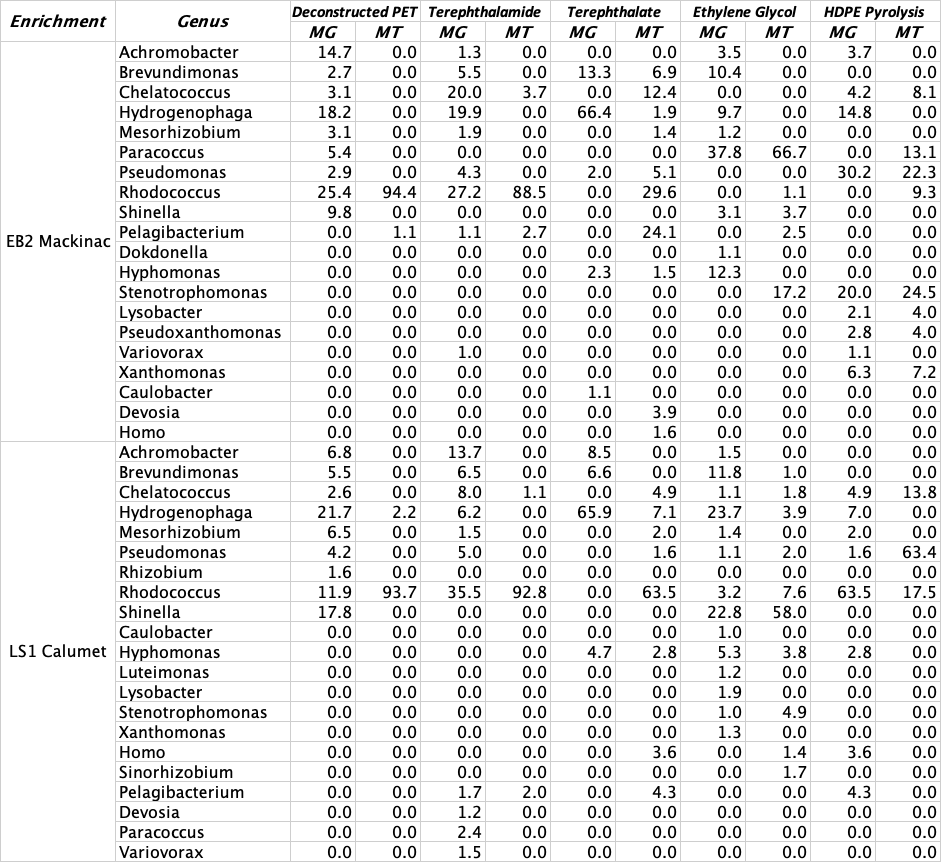


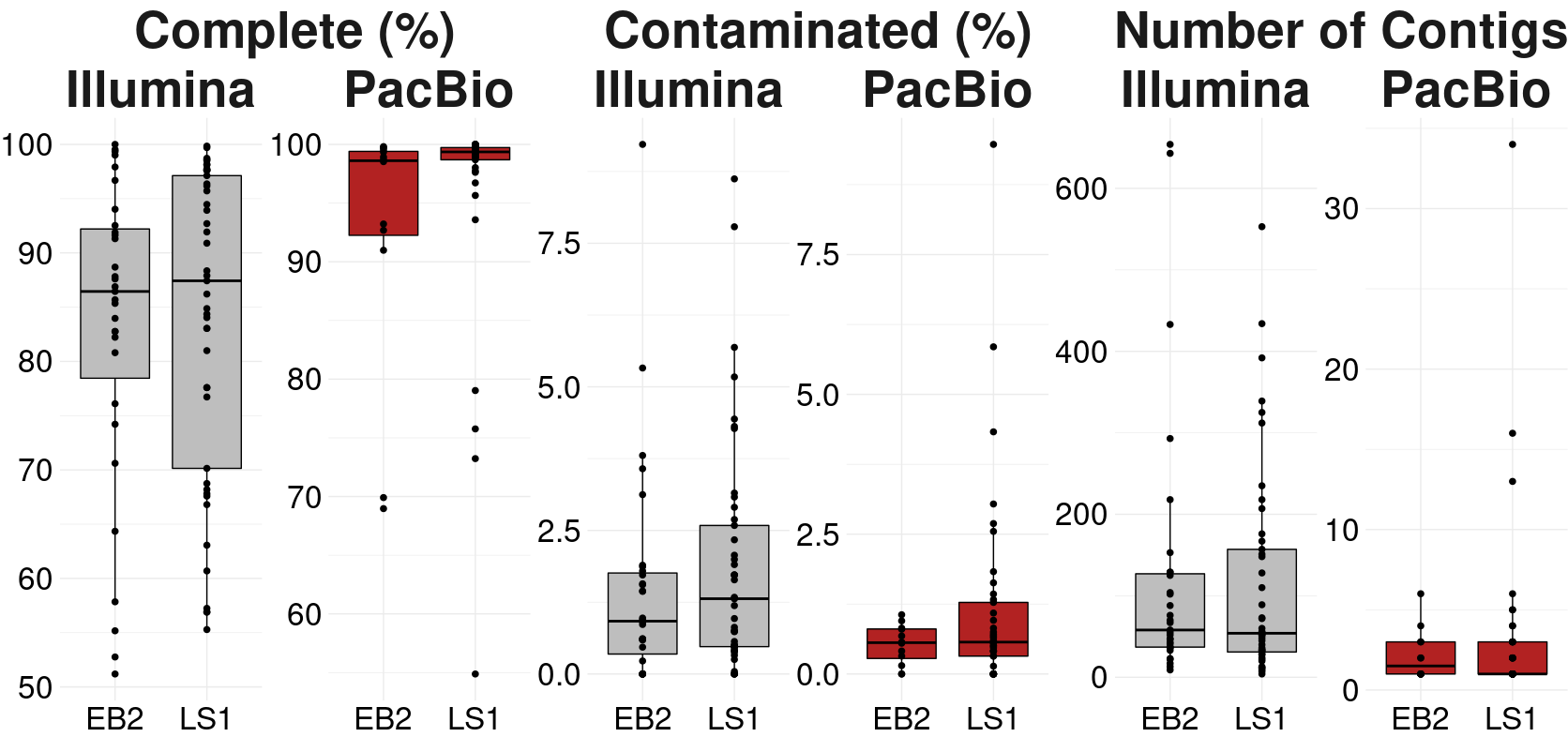


**Figure S4**. Comparison of the completeness, contamination, and number of contigs in Illumina and PacBio MAGs (medium and high quality only). EB2 (EB2_Mackinac), LS1 (LS1_Calumet). Note changing scales on the y-axis.

**Table S8**. Summary of completeness, contamination and number of contigs in short-read (Illumina) and long-read (Pacific Biosciences) MAGs

| ***Enrichment*** | ***Type*** | ***Number of Contigs*** | | | ***Median Contamination*** | ***Median Completeness*** | ***Number of MAGs*** |
| --- | --- | --- | --- | --- | --- | --- | --- |
|  |  | ***Maximum*** | ***Minimum*** | ***Median*** |  |  |  |
| EB2 Mackinac | PacBio | 6 | 1 | 1.5 | 0.56 | 98.6 | 12 |
|  | Illumina | 654 | 9 | 58 | 0.92 | 86.4 | 31 |
| LS2 Calumet | PacBio | 34 | 1 | 1 | 0.57 | 99.4 | 41 |
|  | Illumina | 553 | 4 | 54 | 1.31 | 87.4 | 45 |


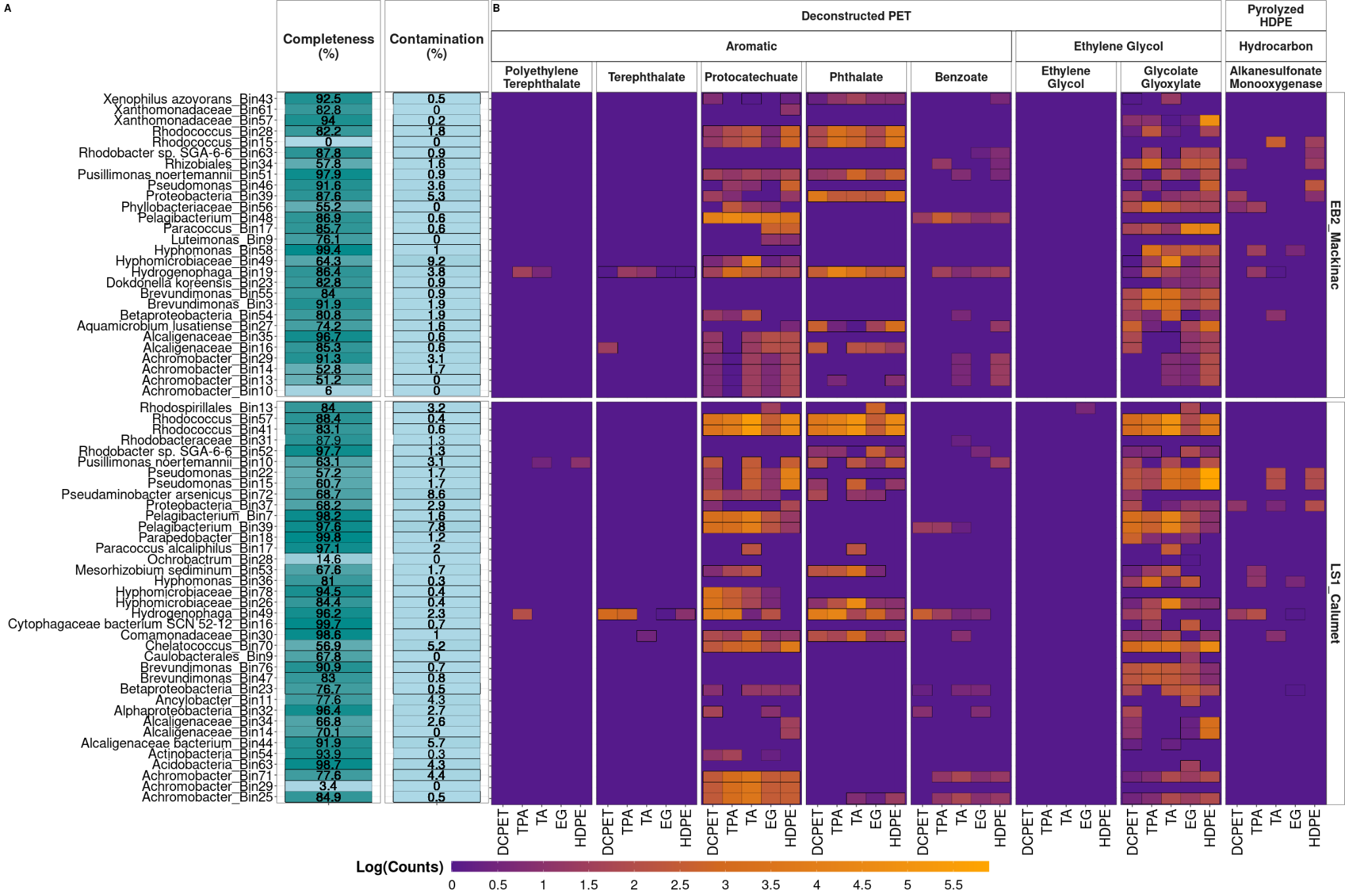


**Figure S5**. (**A**) Completeness and contamination of MAGs obtained from short-read metagenomic sequencing. MAGs with contamination greater than 10% are not shown. (**B**) Gene expression of genes in relevant pathways in each treatment. Numbers shown are the log10 of the sum of htseq counts of genes in each pathway.


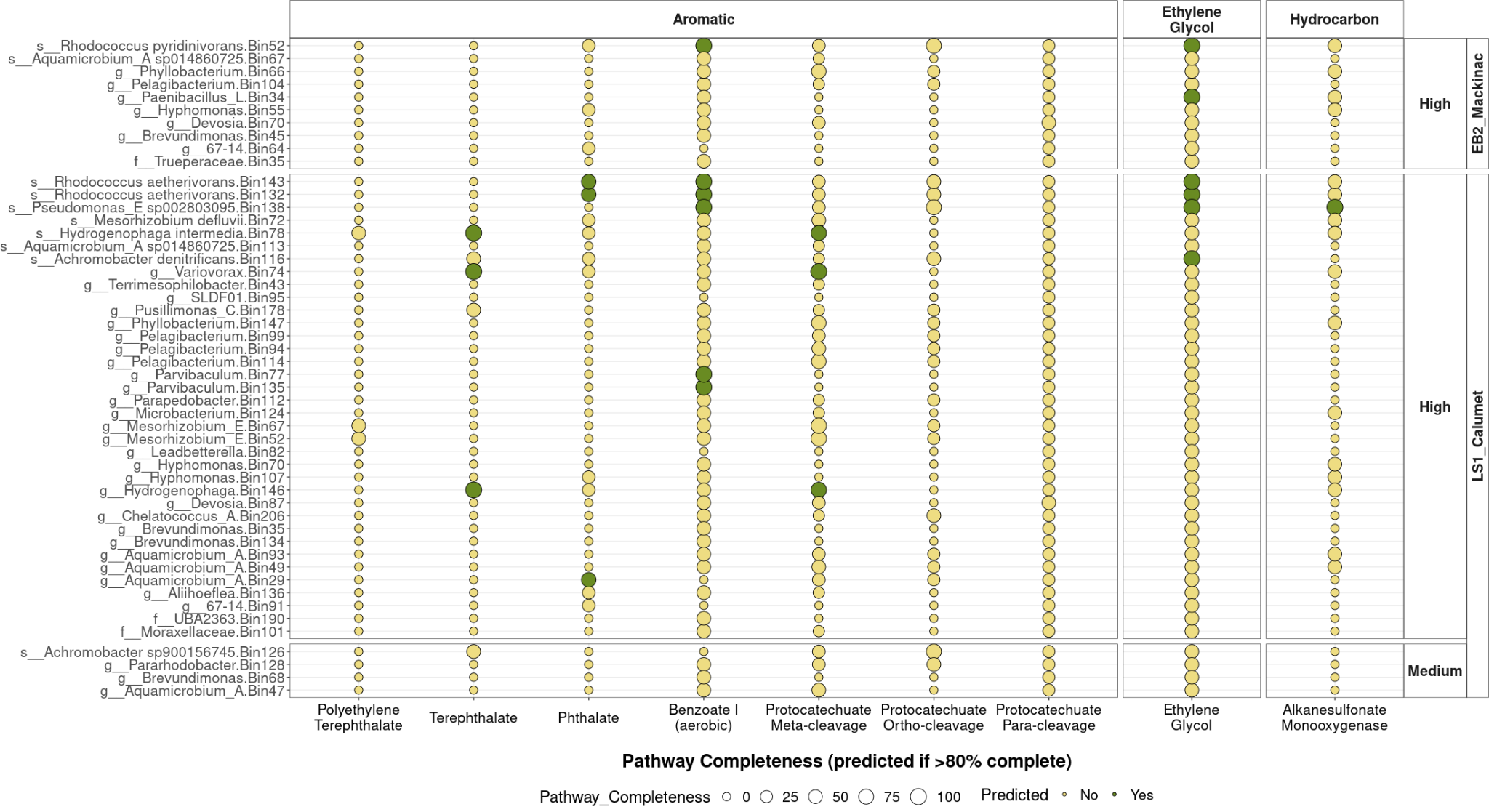


**Figure S6**. Relevant predicted pathways for all MAGs recovered from long-read metagenomic sequencing (Pacific Biosciences). Pathways greater than 80% complete are expected to be functional.

**Table S9**. Summary of high and medium quality MAGs recovered from long-read metagenomic sequencing.


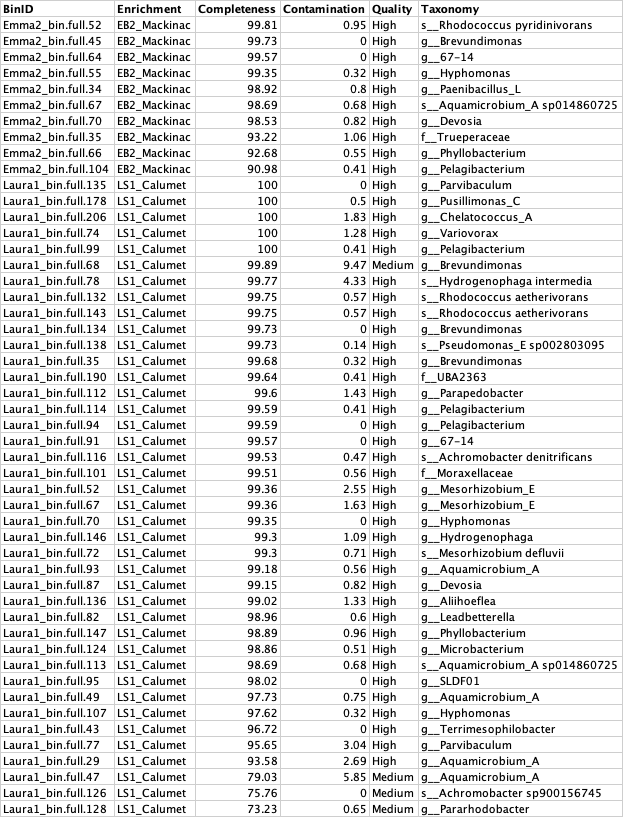


**Table S10**. Summary of high and medium quality MAGs recovered from short-read metagenomic sequencing.


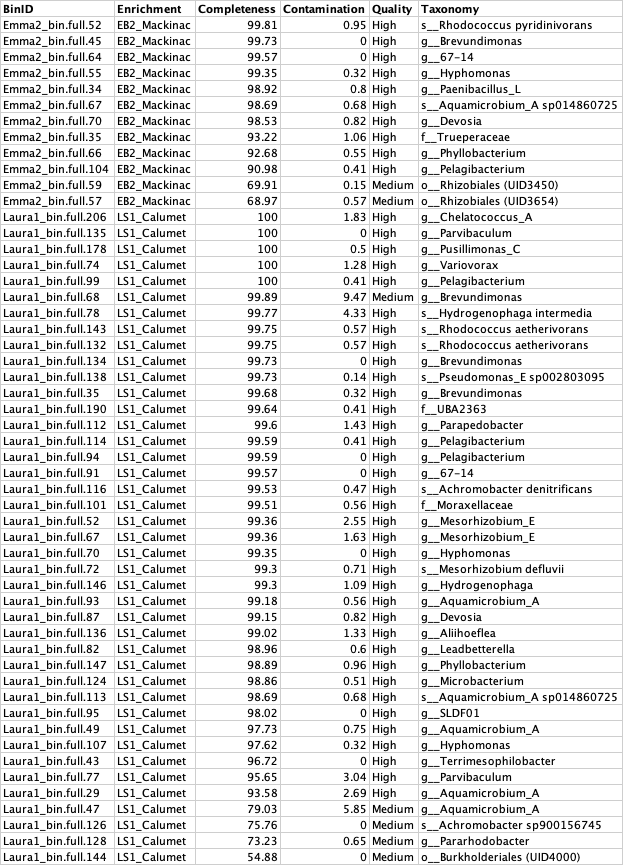

Supplement: Supplementary file 2 — Additional file 1: Table S1. Composition of the chemically deconstructed PET. Table S2. Maximum change in growth (OD600) for each enrichment on each substrate relative to the uninoculated blanks. DPCET (chemically deconstructed PET), TPA (terephthalate), TA (terephthalamide), EG (ethylene glycol), HDPE (pyrolyzed high density polyethylene). Figure S1. Observed alpha diversity of metagenome and metatranscriptome samples. Sequence type abbreviations: MT (metatranscriptomic) or MG (metagenomic). Substrate abbreviations: high density polyethylene pyrolysis (HDPE), deconstructed PET (DCPET), terephthalate (TPA), terephthalamide (TA), and ethylene glycol (EG). Figure S2. Shannon alpha diversity of metagenome and metatranscriptome samples. Sequence type abbreviations: MT (metatranscriptomic) or MG (metagenomic). Substrate abbreviations: high density polyethylene pyrolysis (HDPE), deconstructed PET (DCPET), terephthalate (TPA), terephthalamide (TA), and ethylene glycol (EG). Table S3. Shannon and Observed alpha diversity of metagenomic (MG) and metatranscriptomic (MT) samples. Substrate abbreviations: high density polyethylene pyrolysis (HDPE), deconstructed PET (DCPET), terephthalate (TPA), terephthalamide (TA), and ethylene glycol (EG). Table S4. Kruskal-Wallis comparison of alpha diversity between sequence types (metagenome versus metatranscriptome). Figure S3. Bray Curtis principal coordinates analysis (PCoA). Shapes denote sequence type: MT (metatranscriptomic) or MG (metagenomic). Substrate abbreviations: high density polyethylene pyrolysis (HDPE), deconstructed PET (DCPET), terephthalate (TPA), terephthalamide (TA), and ethylene glycol (EG). Table S5. Results summary of PERMANOVA comparison of microbial community composition between sequence type (metagenomic versus metatranscriptomic samples). Table S6. Results summary of PERMANOVA comparison of microbial community composition between aromatic metatranscriptomic samples (deconstructed PET, terephthalate, and terepht [file 40168_2023_1645_MOESM1_ESM.docx]
